# Publisher Correction: Parallel evolution in the emergence of highly pathogenic avian influenza A viruses

**DOI:** 10.1038/s41467-020-20006-5

**Published:** 2020-11-23

**Authors:** Marina Escalera-Zamudio, Michael Golden, Bernardo Gutiérrez, Julien Thézé, Jeremy Russell Keown, Loic Carrique, Thomas A. Bowden, Oliver G. Pybus

**Affiliations:** 1 Department of Zoology, Oxford University, Parks Rd, Oxford, OX1 3PS UK; 2 Division of Structural Biology, Wellcome Centre for Human Genetics, Oxford, OX3 7BN UK; 3 Department of Pathobiology and Population Sciences, Royal Veterinary College, London, UK

**Keywords:** Molecular evolution, Phylogenetics, Influenza virus, Viral evolution

Correction to: *Nature Communications* 10.1038/s41467-020-19364-x, published online 2 November 2020.

The original version of this Article was updated shortly after publication, because the Supplementary Information file was the incorrect version. The error has now been fixed and the correct Supplementary Information PDF is available to download from the HTML version of the Article and can be found as Supplementary Information to this Article.

