# Supplementary material for: Parallel evolution in the emergence of highly pathogenic avian influenza A viruses
Source: Nat Commun. 2020 Nov 2;11:5511. doi: 10.1038/s41467-020-19364-x (PMC7608645; doi:10.1038/s41467-020-19364-x)
Supplement: Supplementary file 3 — Description of Additional Supplementary Files [file 41467_2020_19364_MOESM3_ESM.pdf]

Description of Additional Supplementary Files

Supplementary Data 1.
Description: Accession numbers for reduced datasets
H5_H7_ACCESSIONS.xls

Supplementary Data 2.
Description: ML trees for the reconstructions of ancestral states
RASML_H5_H7.pdf

Supplementary Data 3.
Description: HA large-scale phylogenetic trees coloured according to amino acid states/ phenotype.
LARGE_SCALE_H5_H7.pdf

Supplementary Data 4.
Description: Likelihood Ratio Tests and PSS scored under BST
H5_H7_LRT_PSS_CODEML.xls
