# Supplementary material for: Parallel evolution in the emergence of highly pathogenic avian influenza A viruses
Source: Nat Commun. 2020 Nov 2;11:5511. doi: 10.1038/s41467-020-19364-x (PMC7608645; doi:10.1038/s41467-020-19364-x)
Supplement: Supplementary file 5 — Supplementary Data 2 [file 41467_2020_19364_MOESM5_ESM.pdf]

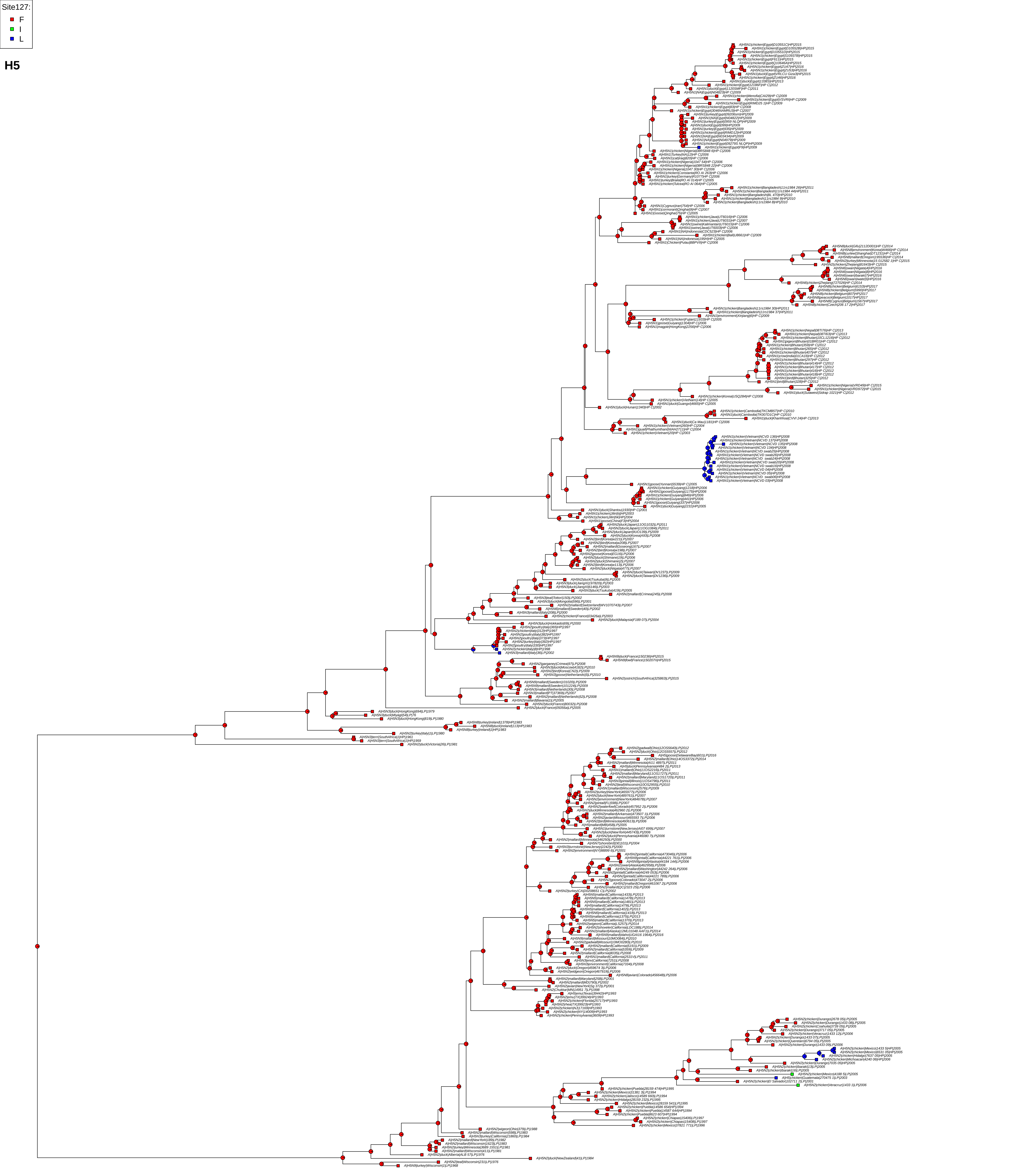
Site127:
F
I
L
H5

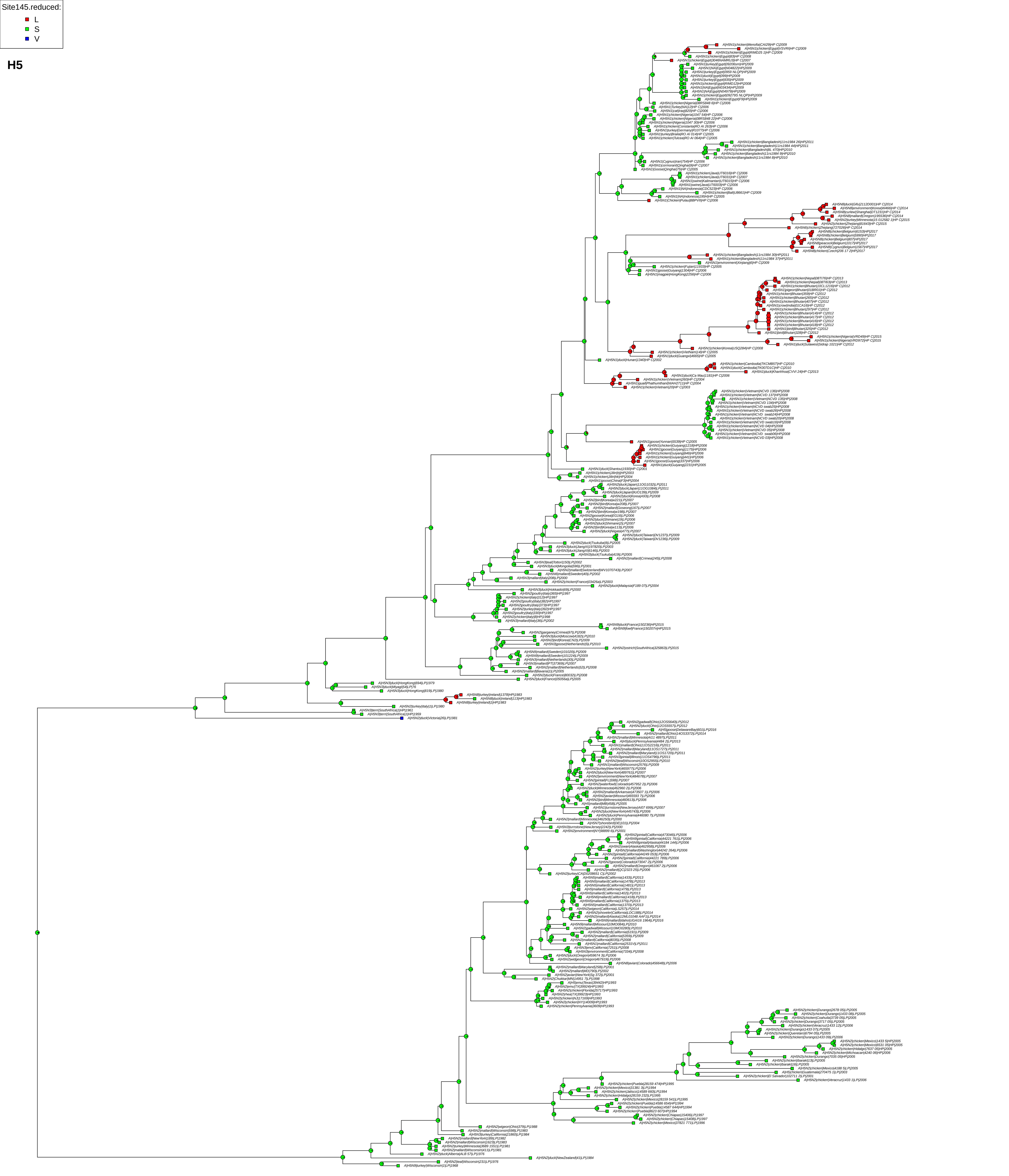
Site145.reduced:
L
S
V
H5

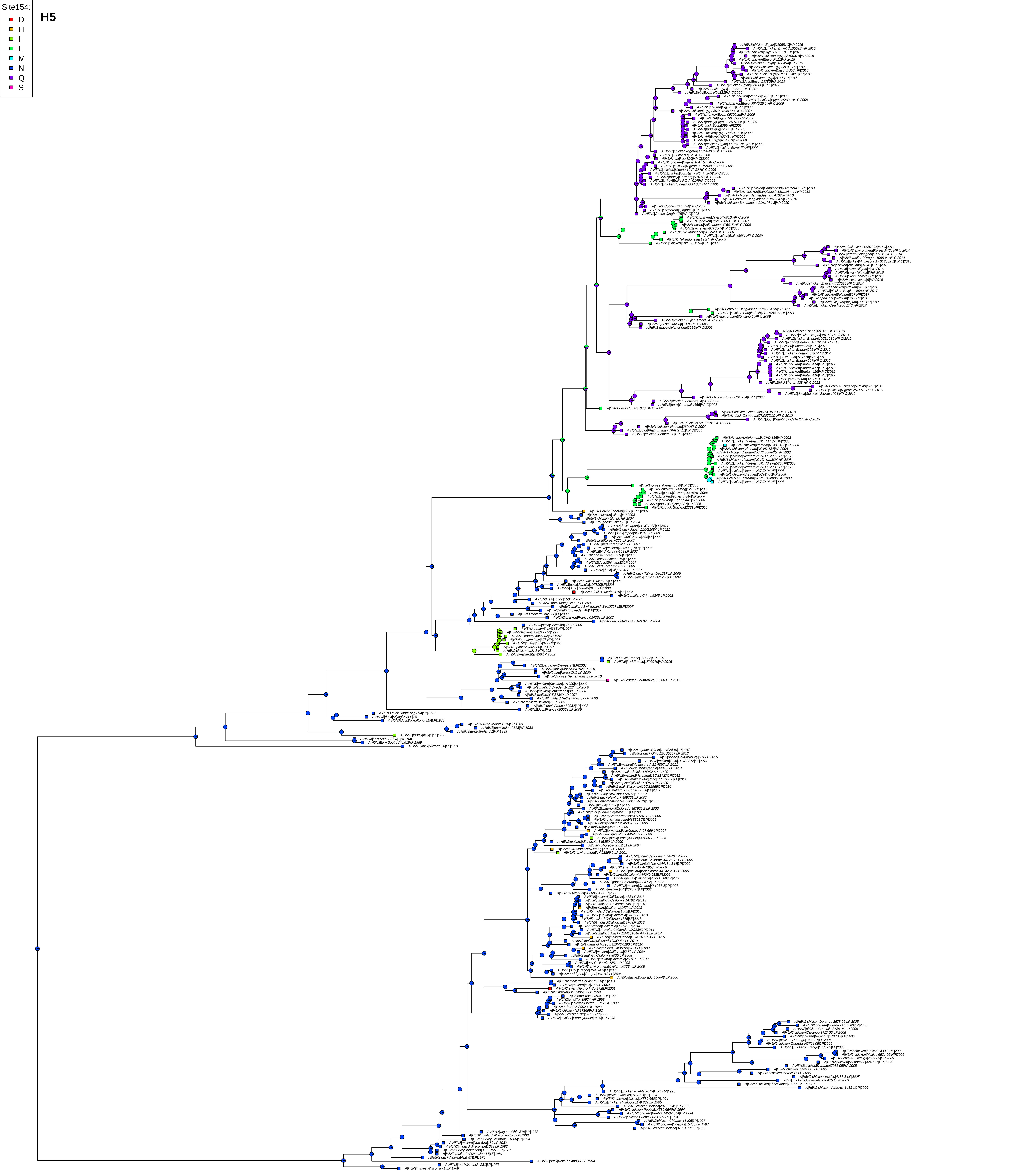
H5
Site154:
D
H
I
L
M
N
Q
S

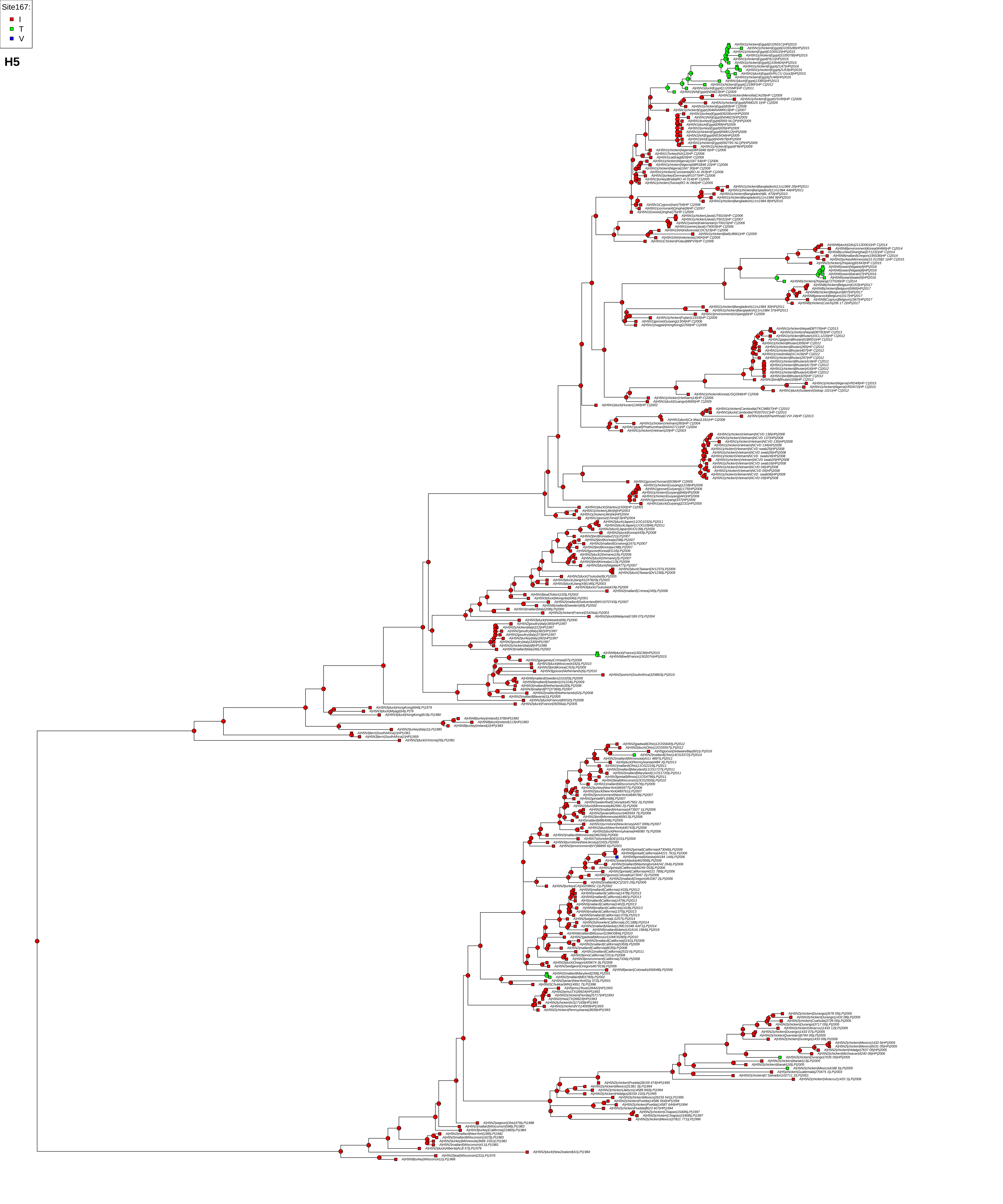
Site167:
I
T
V
H5

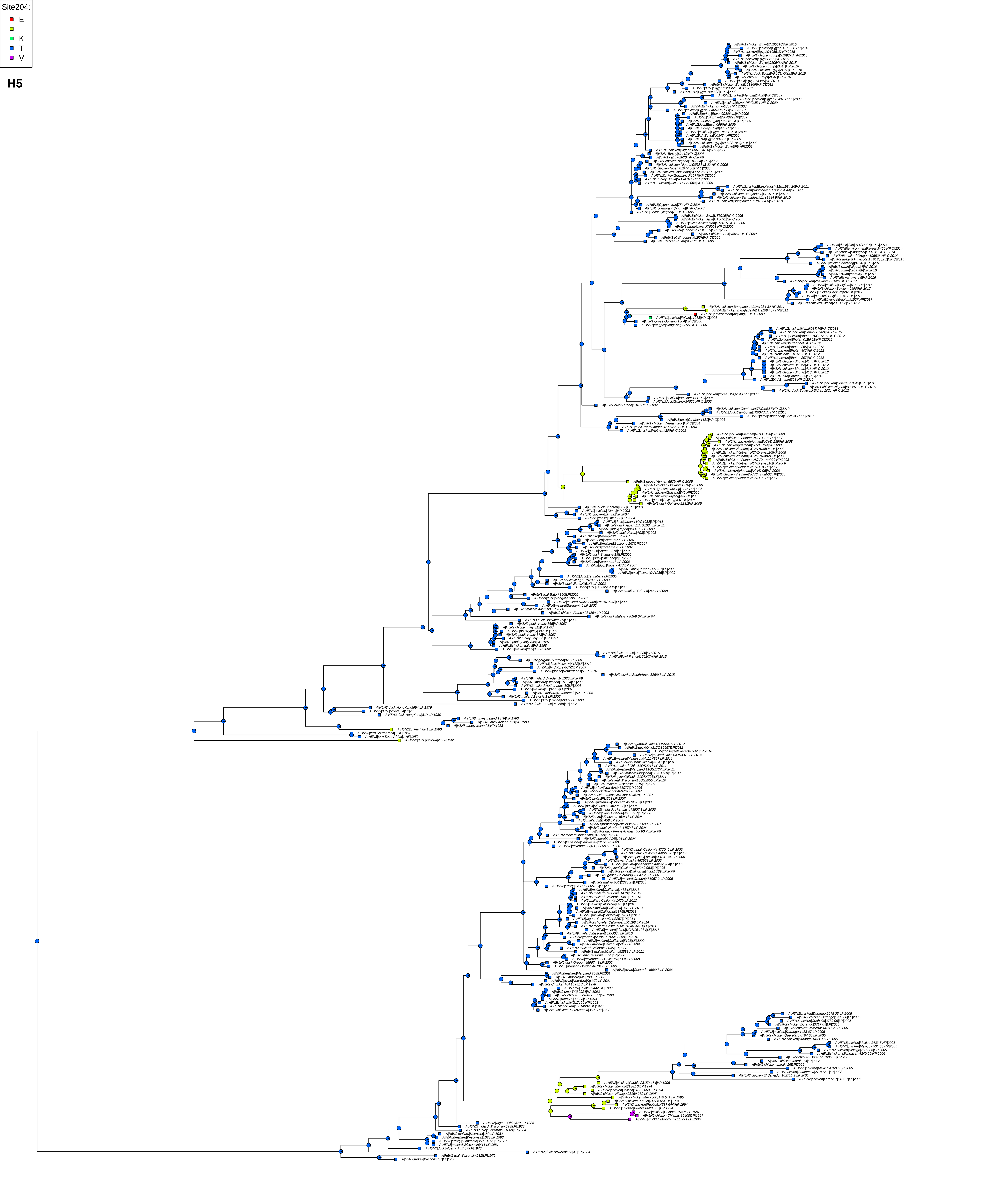

Site204:
E
I
K
T
V
H5

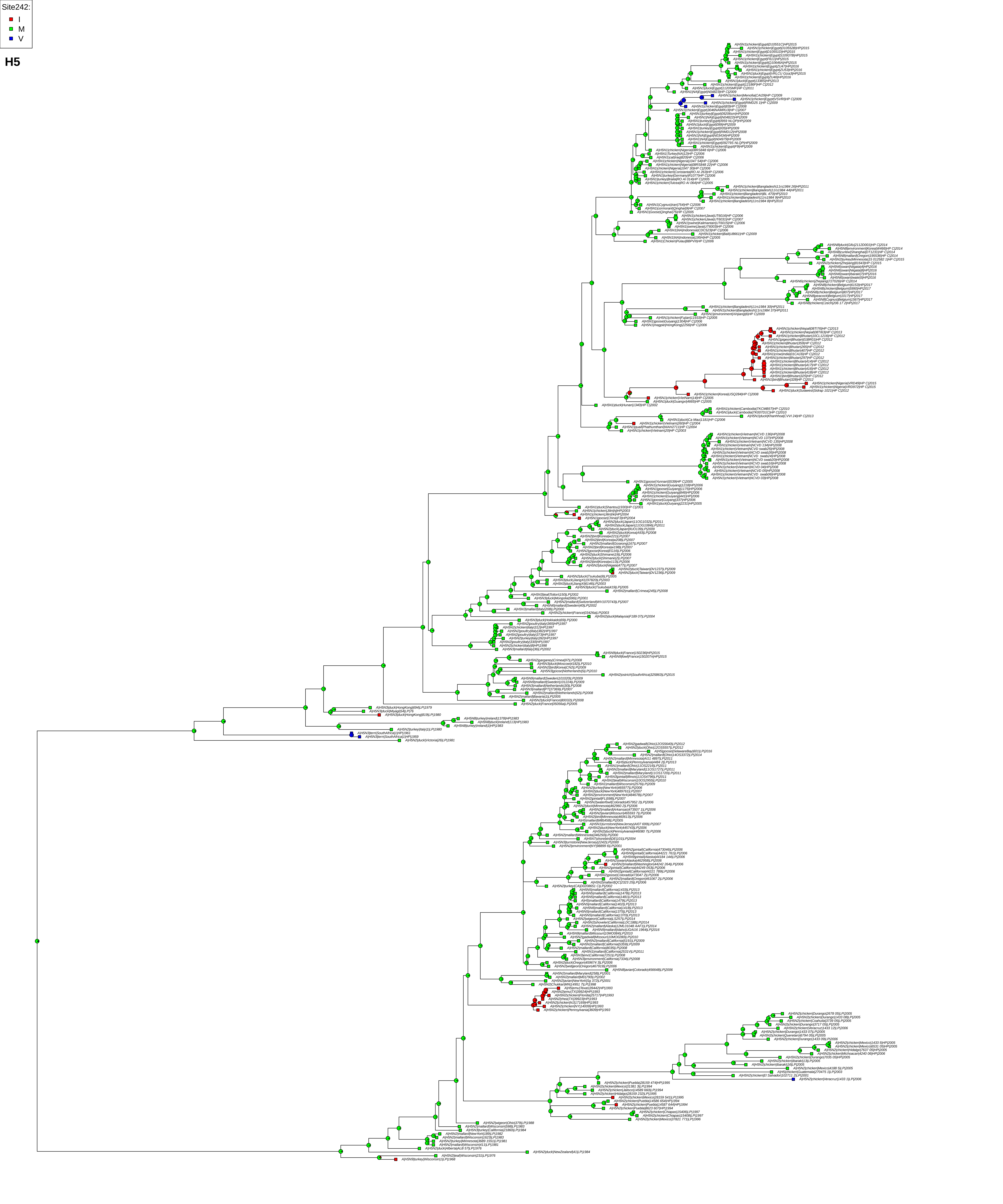
Site242:
I
M
V
H5

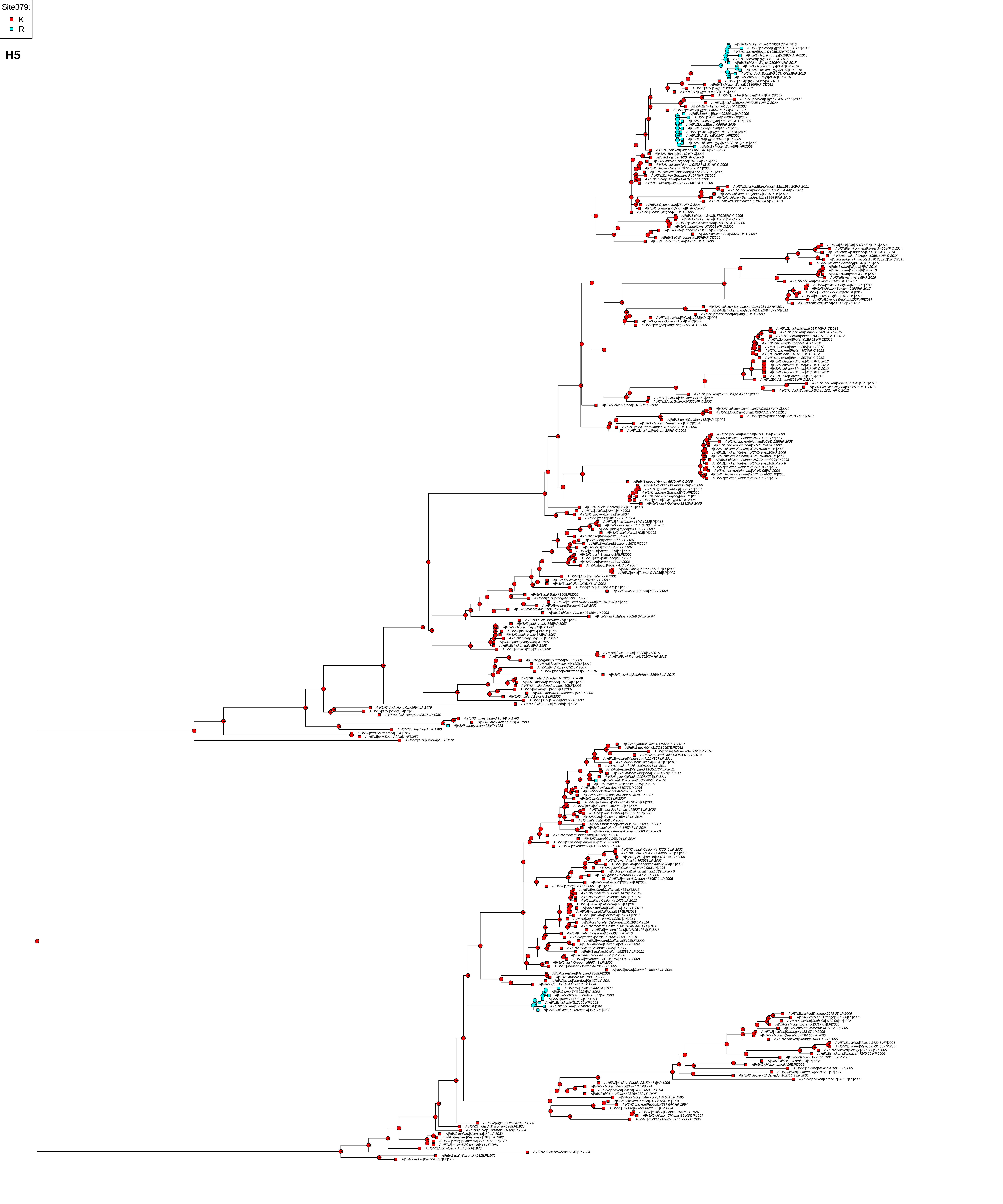

Site379:
K
R
H5

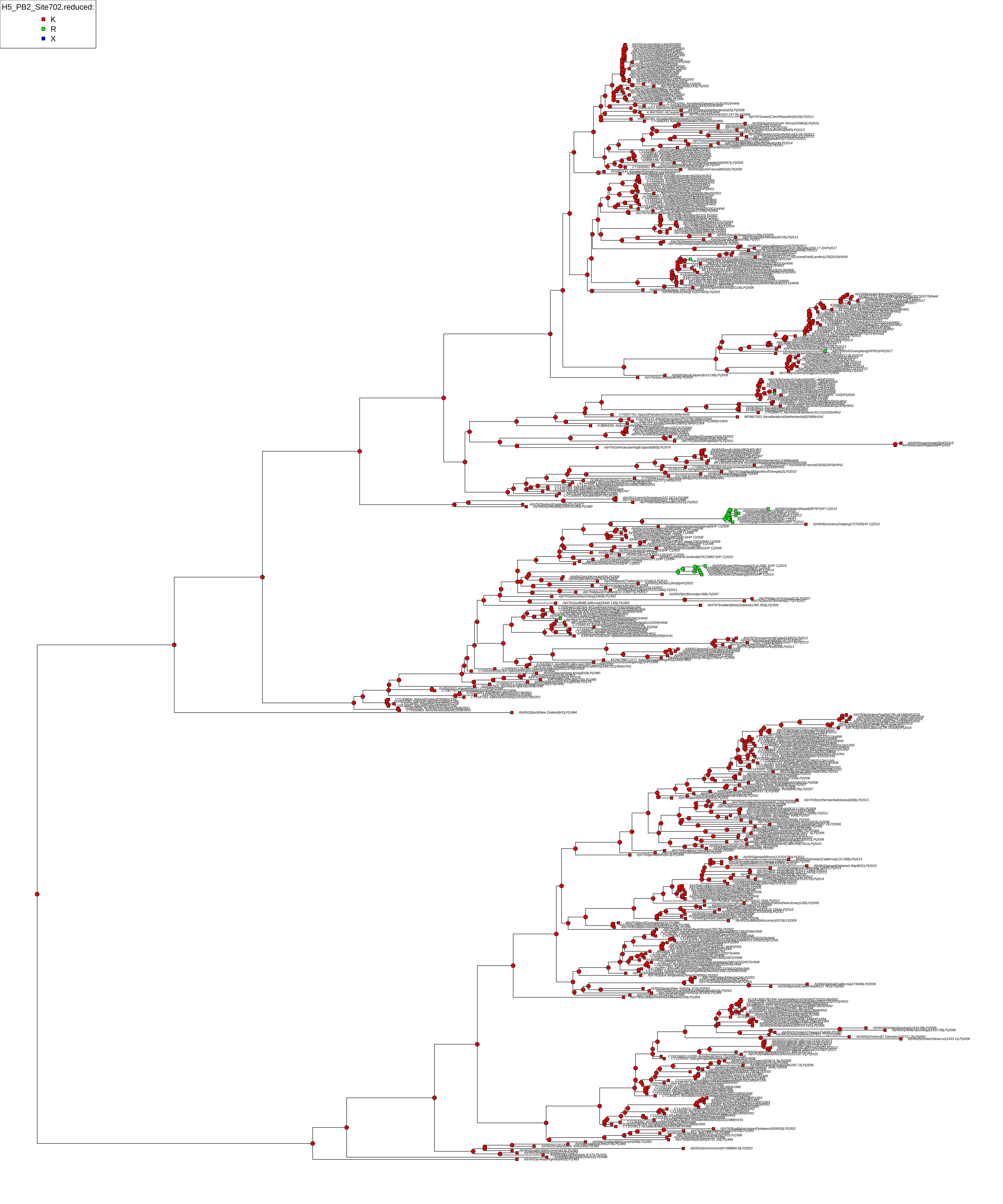
H5_PB2_Site702.reduced:
K
R
X

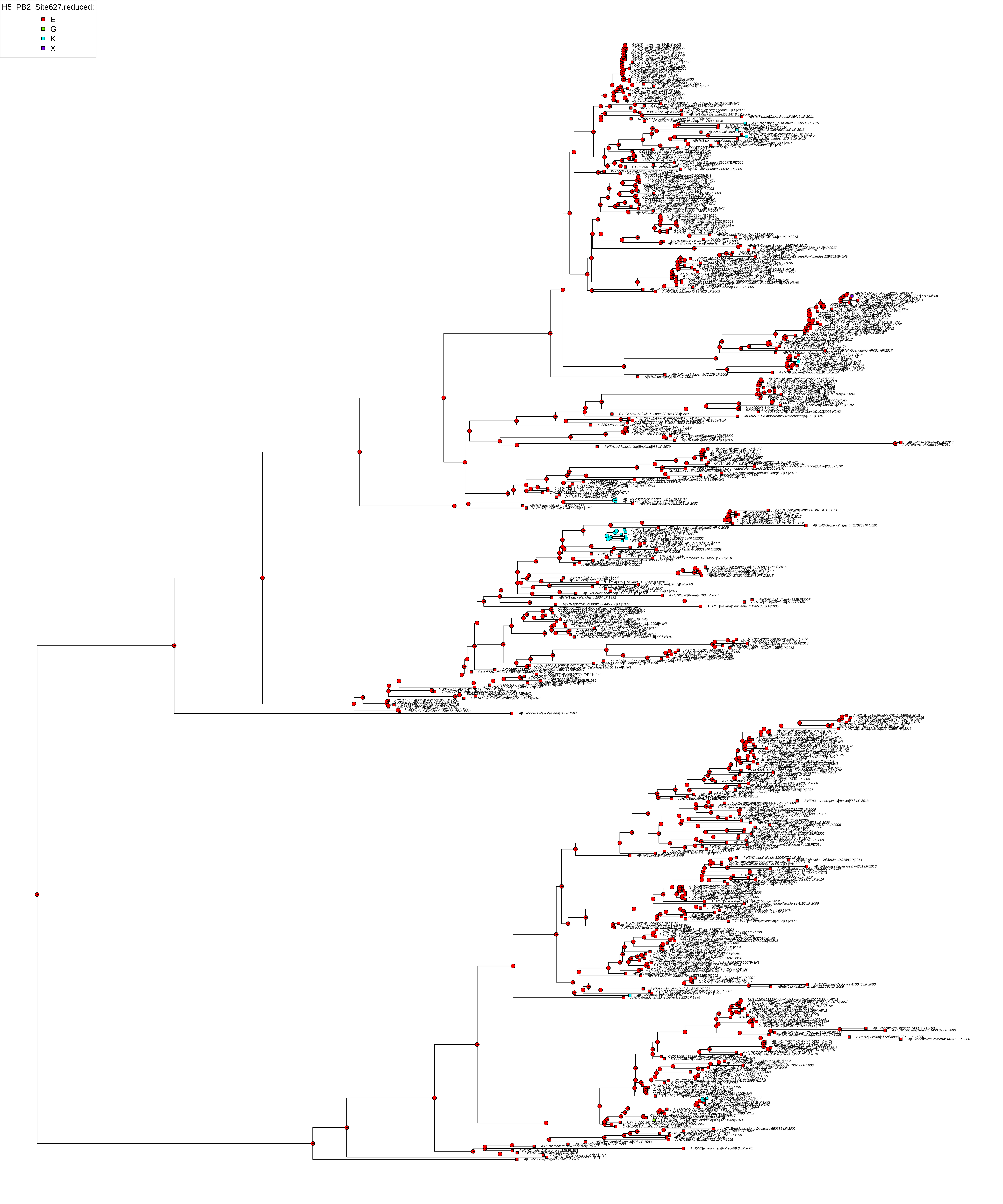
H5_PB2_Site627.reduced:
E
G
K
X

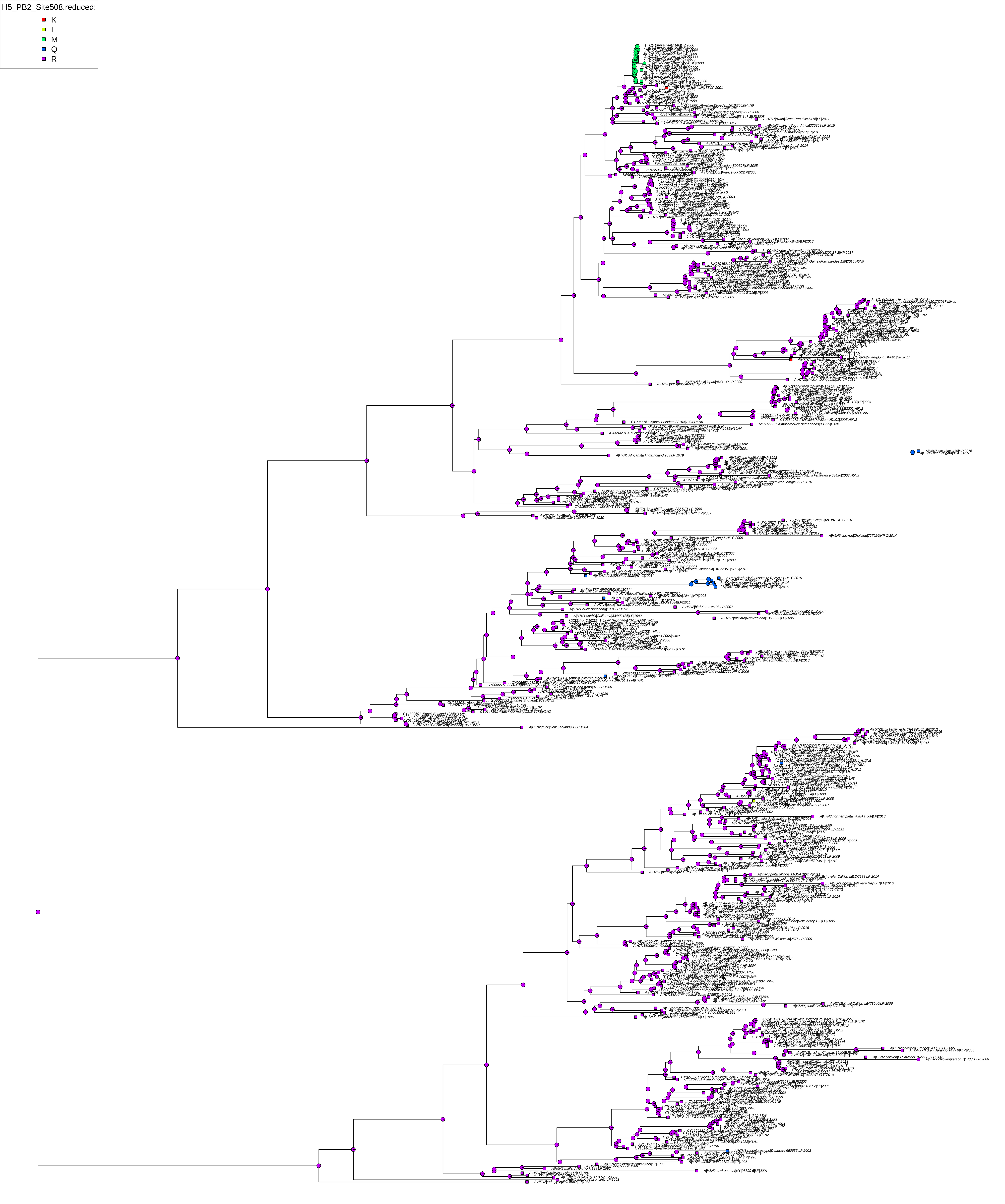
H5_PB2_Site508.reduced:
K
L
M
Q
R

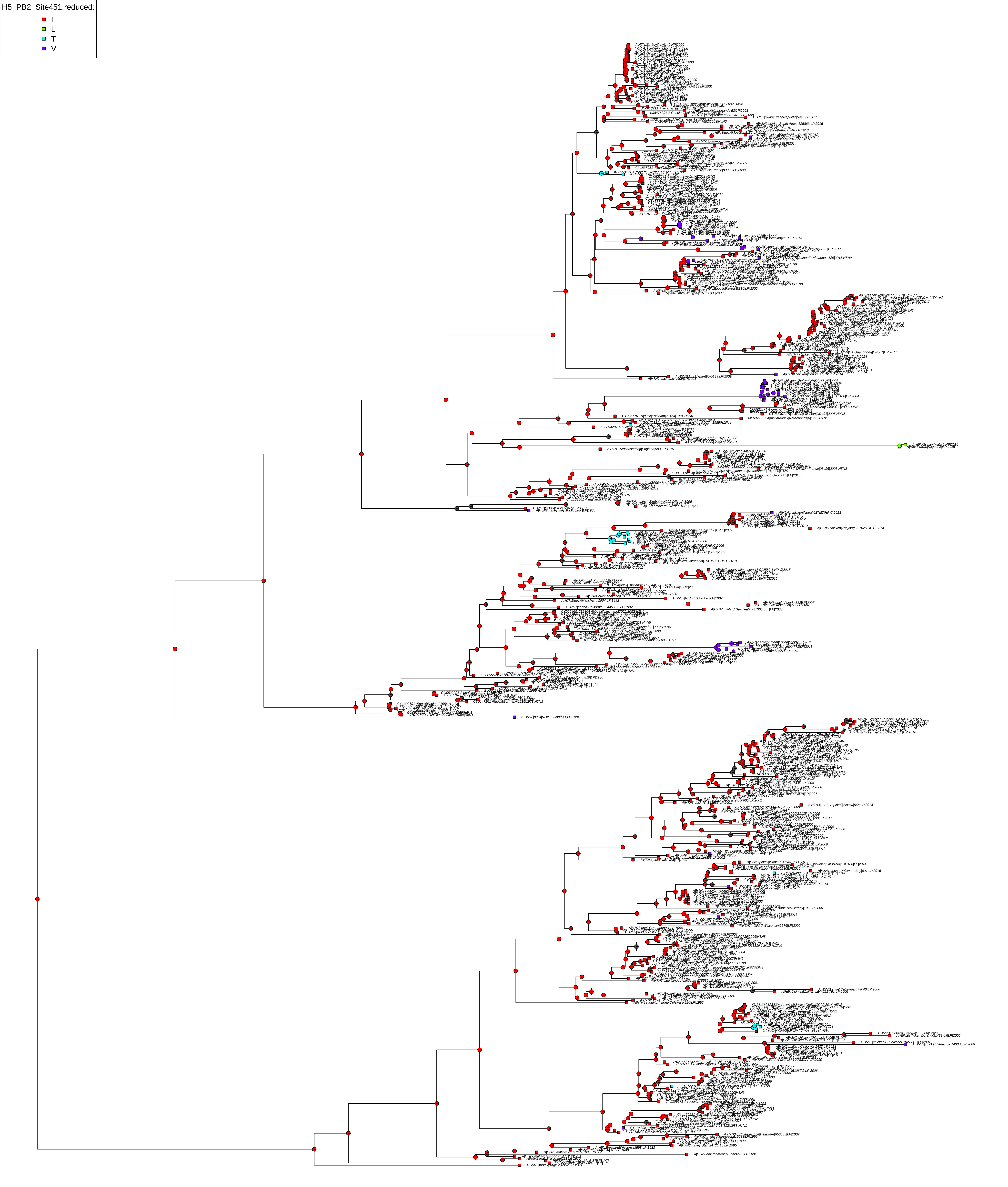
H5_PB2_Site451.reduced:
I
L
T
V

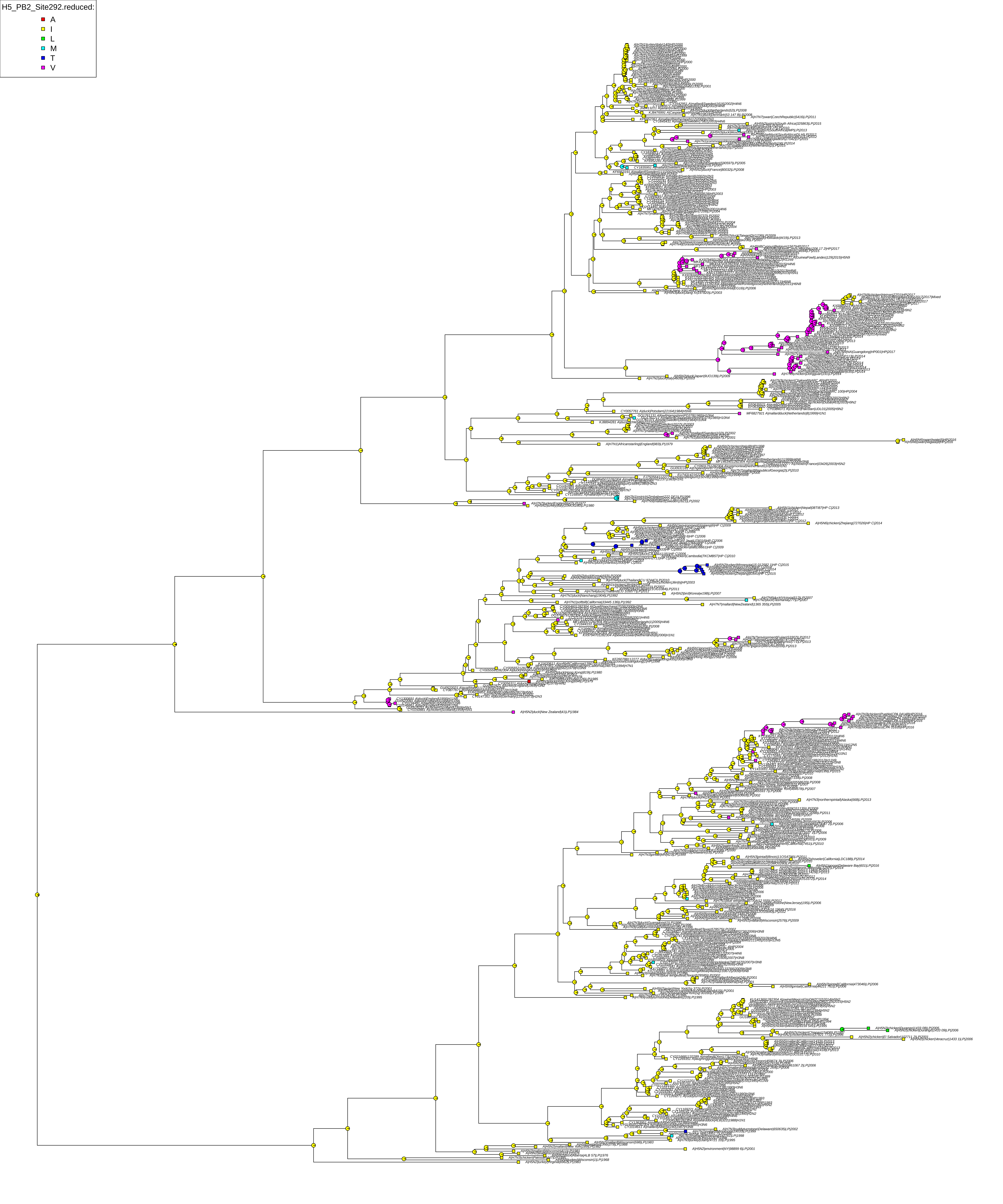
H5_PB2_Site292.reduced:
A
I
L
M
T
V

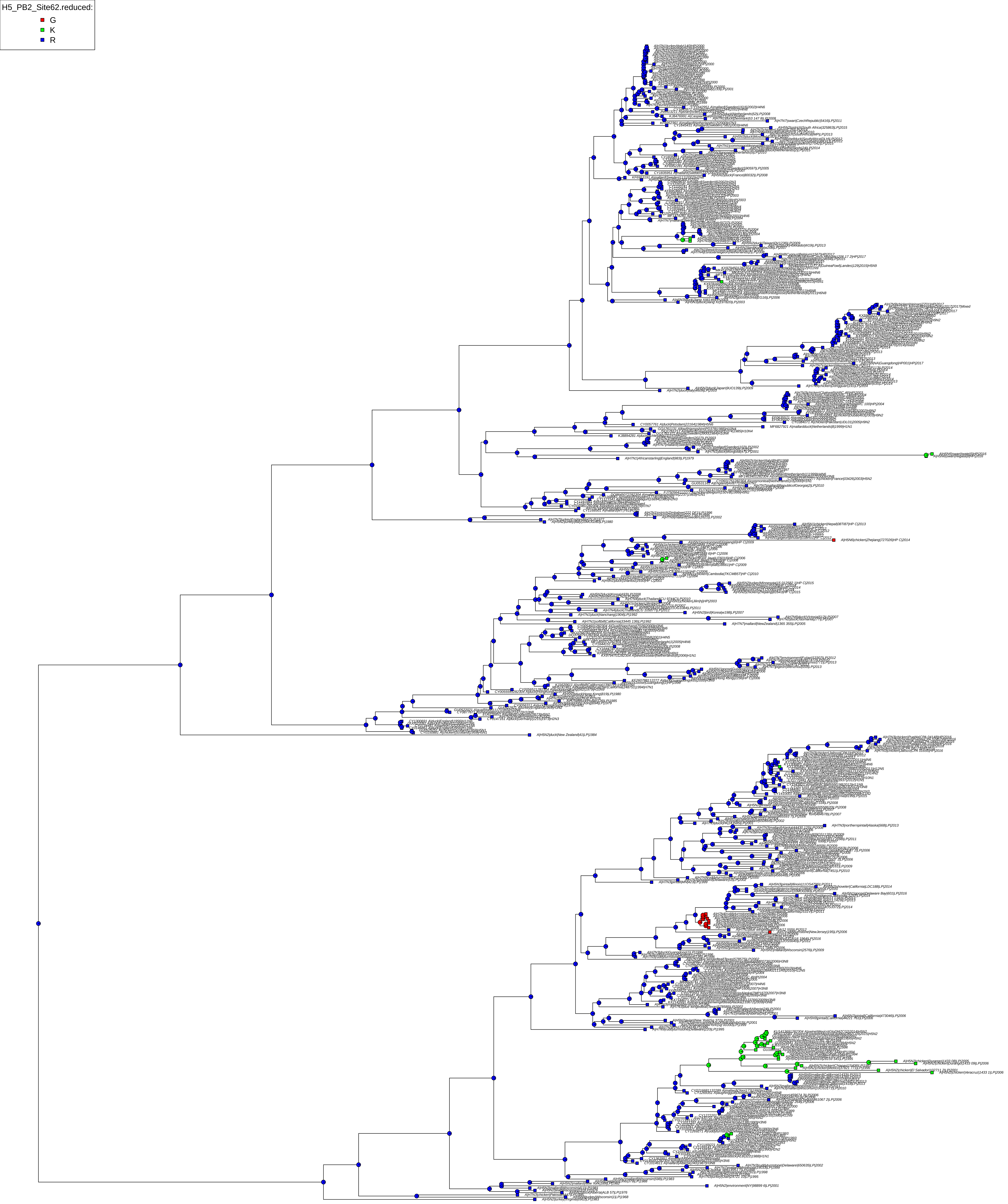
H5_PB2_Site62.reduced:
G
K
R

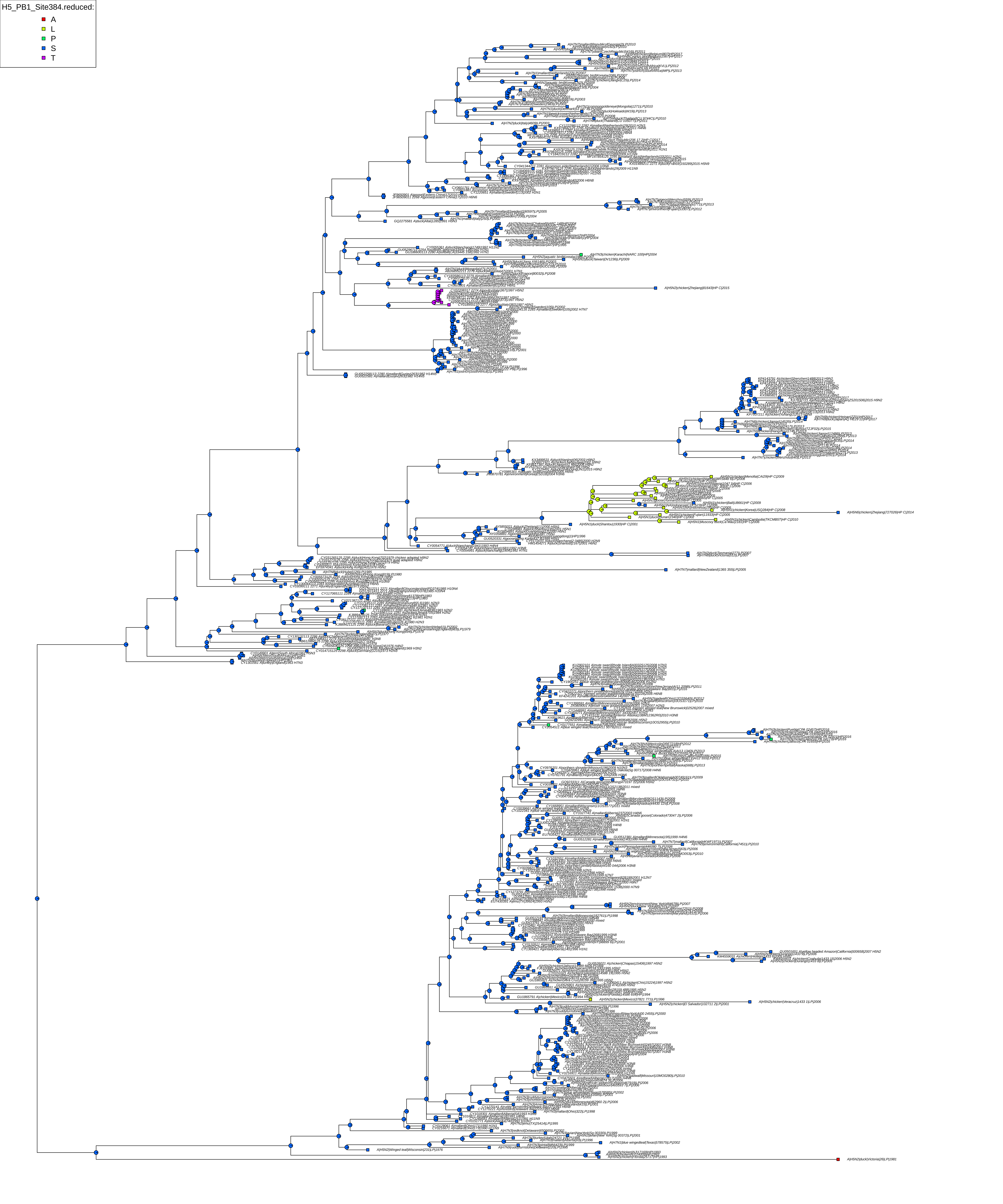
H5_PB1_Site384.reduced:
A
L
P
S
T

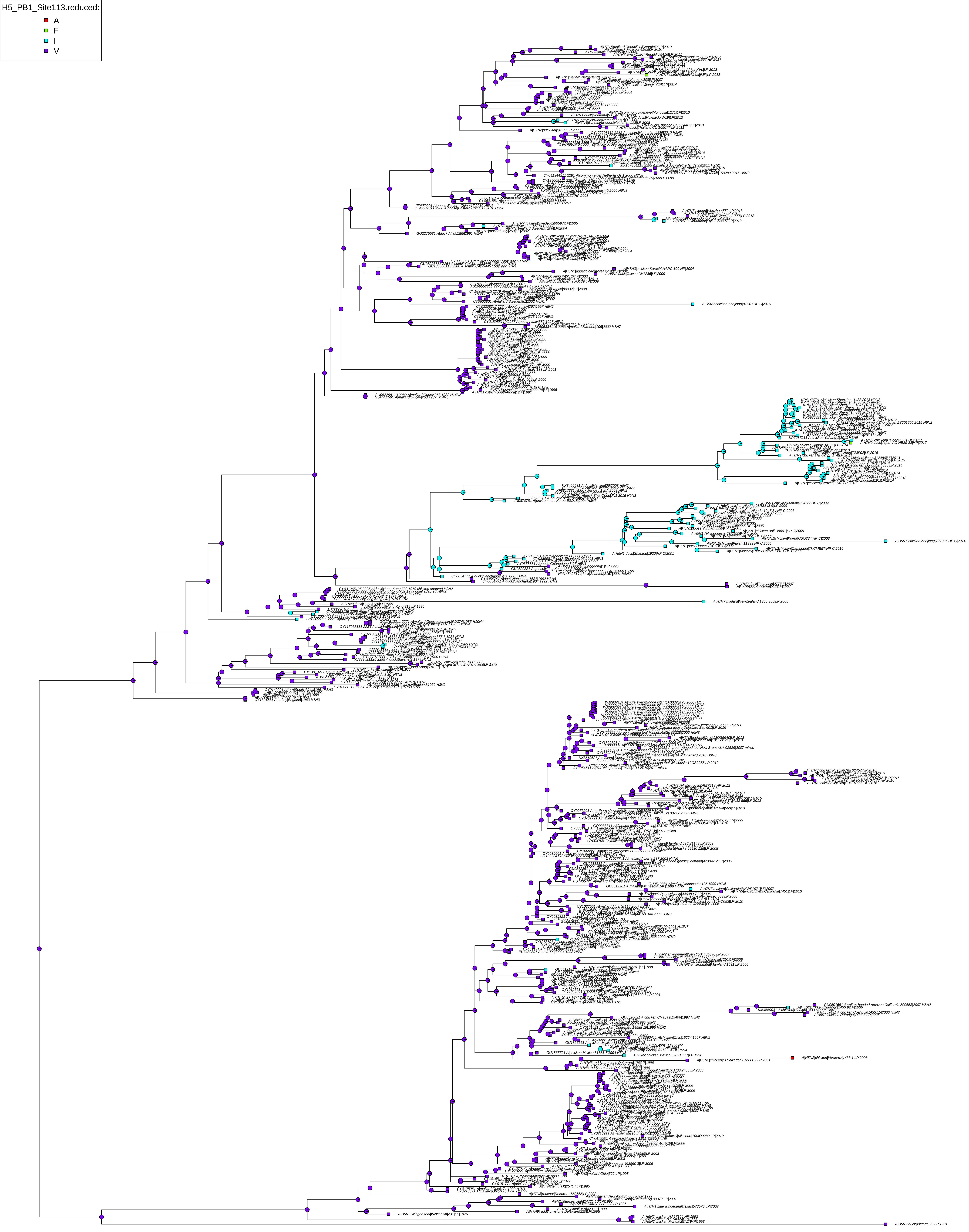
H5_PB1_Site113.reduced:
A
F
I
V

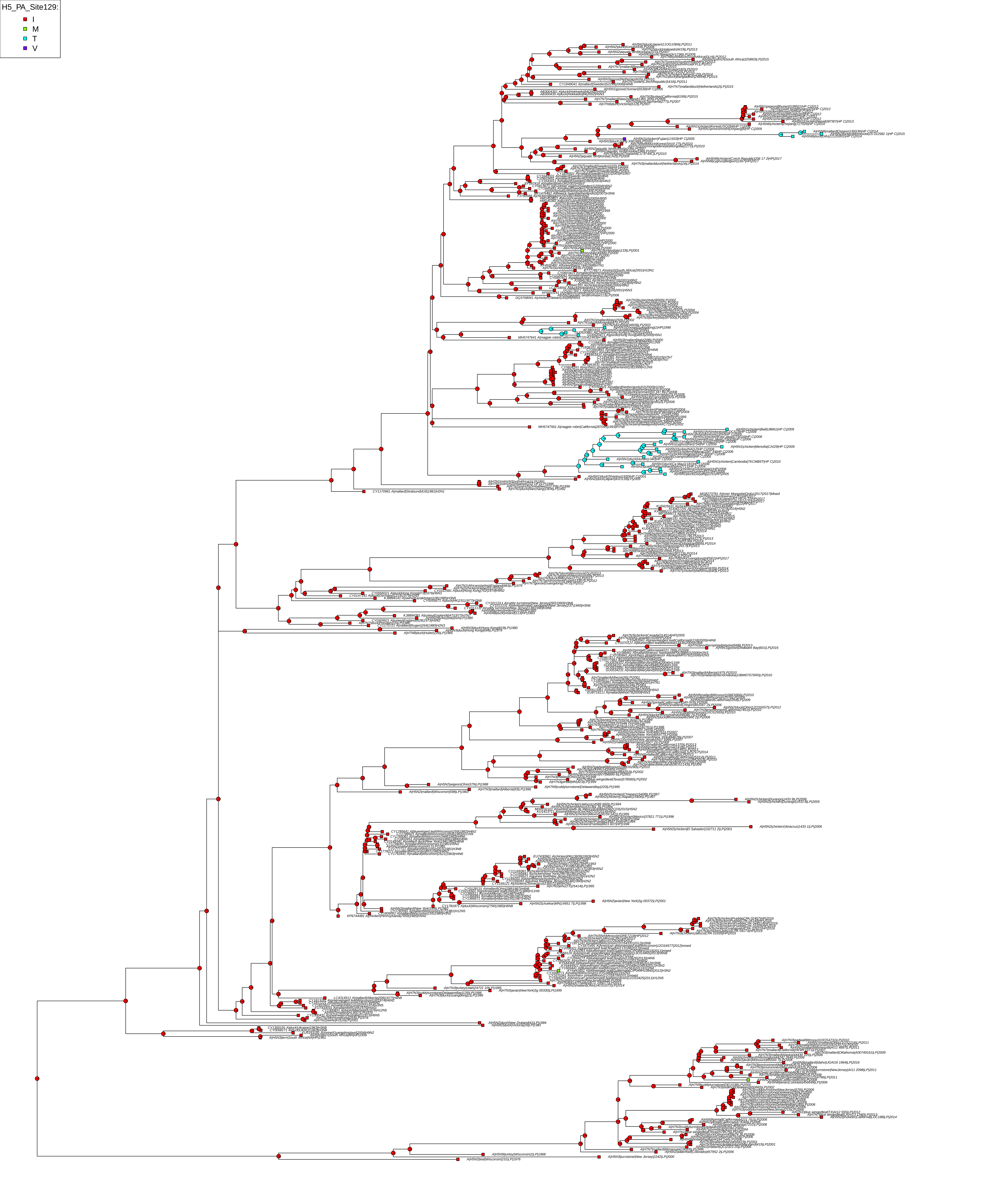
H5_PA_Site129:
I
M
T
V

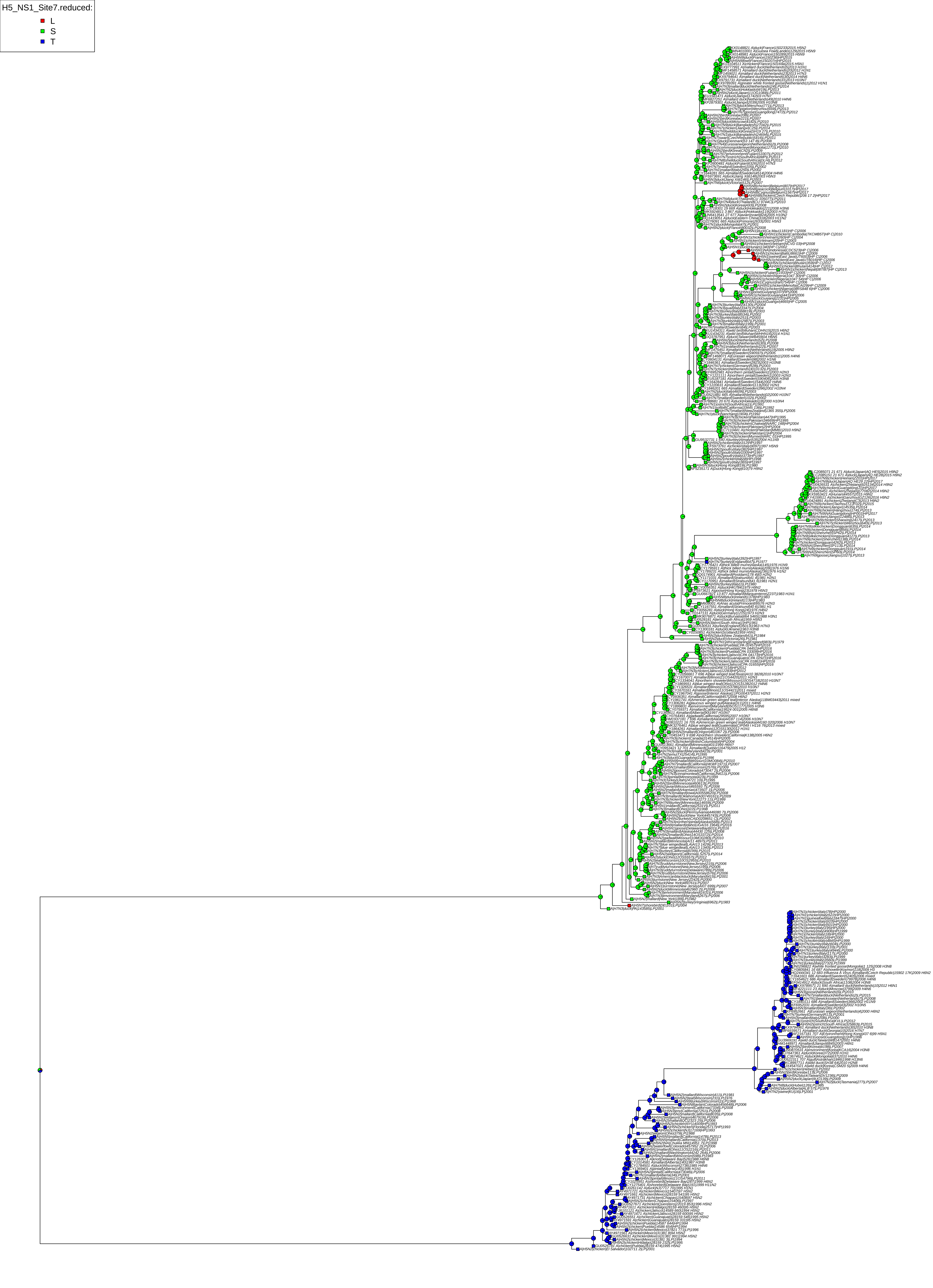
H5_NS1_Site7.reduced:
L
S
T

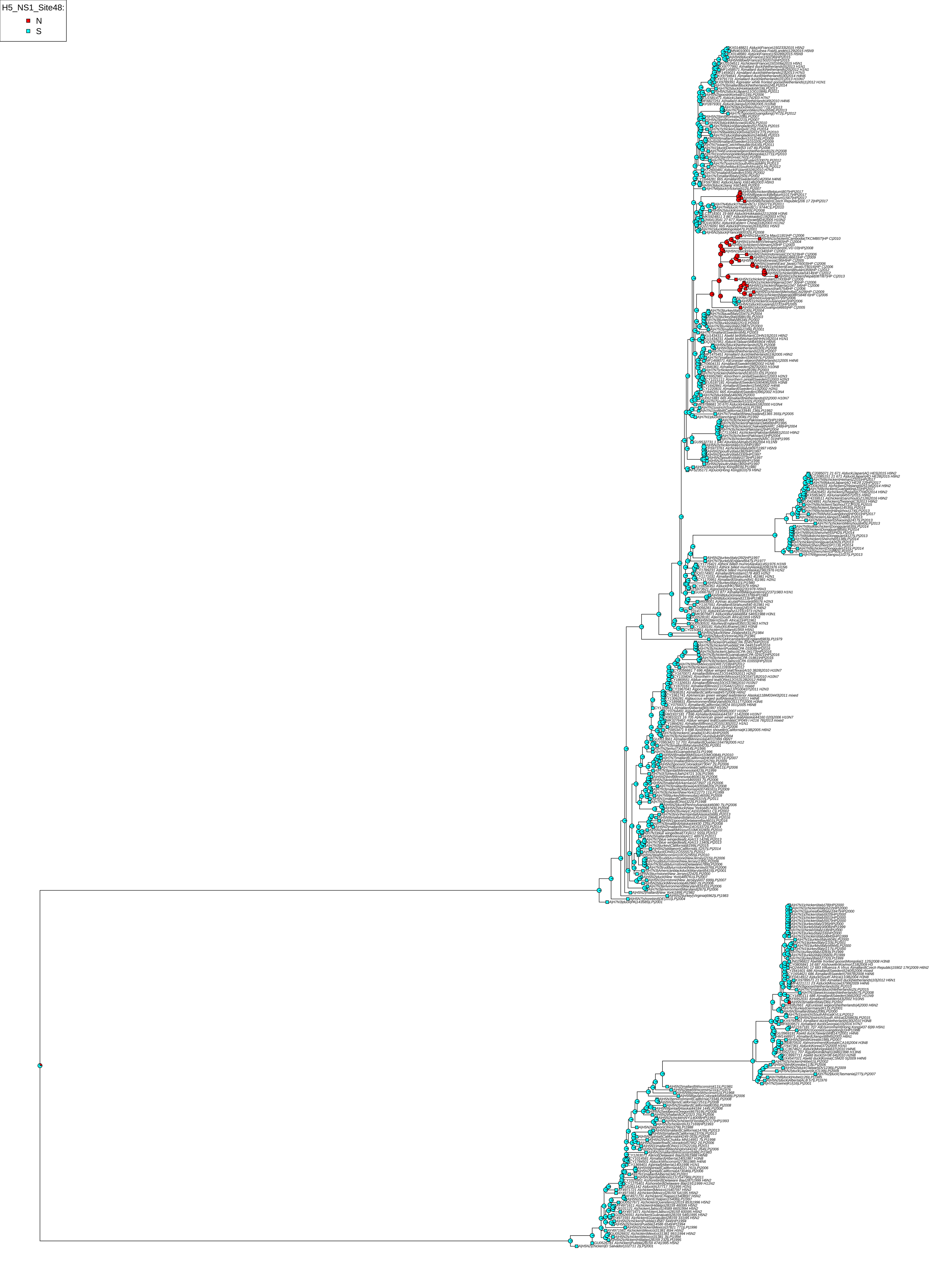
H5_NS1_Site48:
N
S

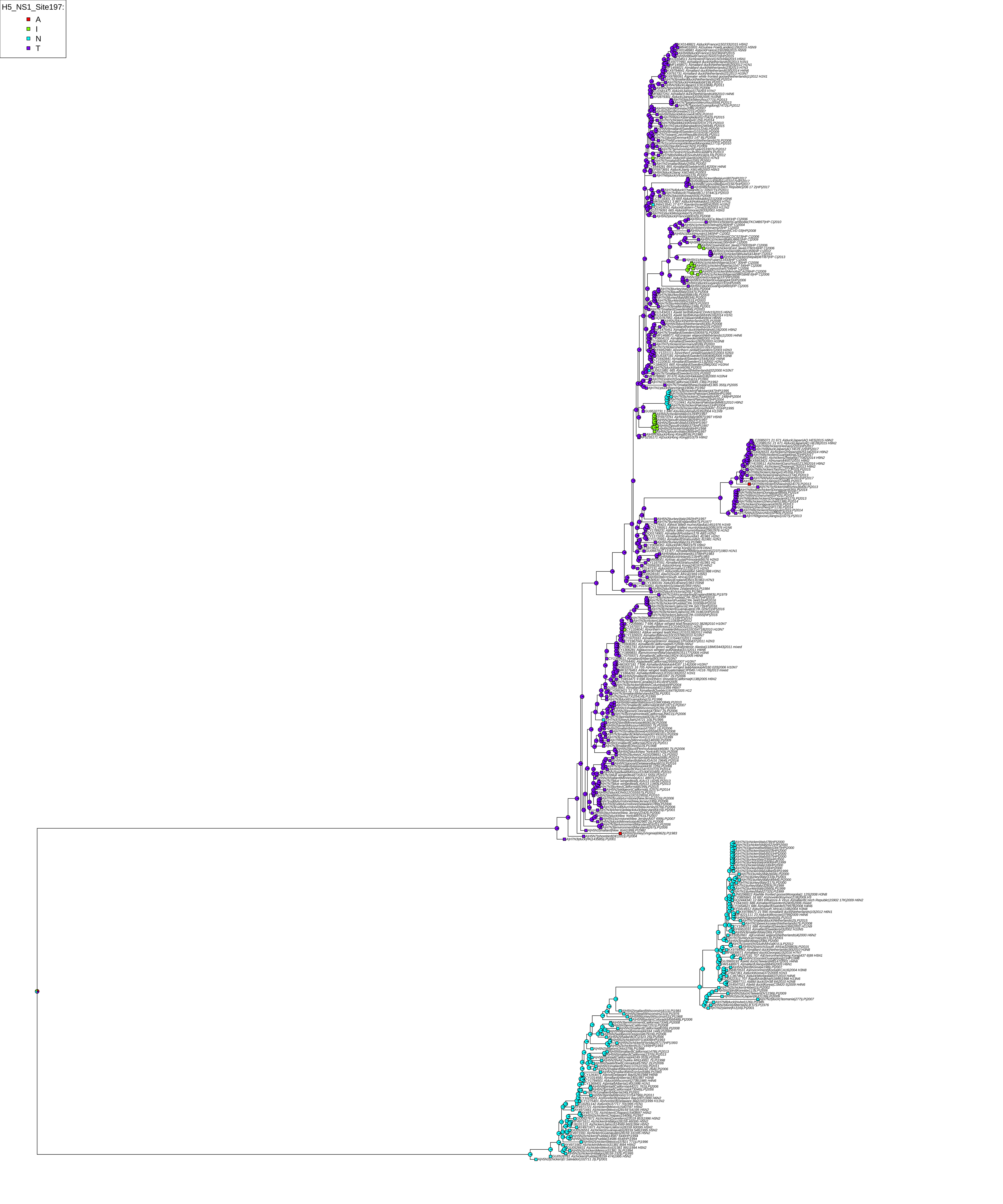
H5_NS1_Site197:
A
I
N
T

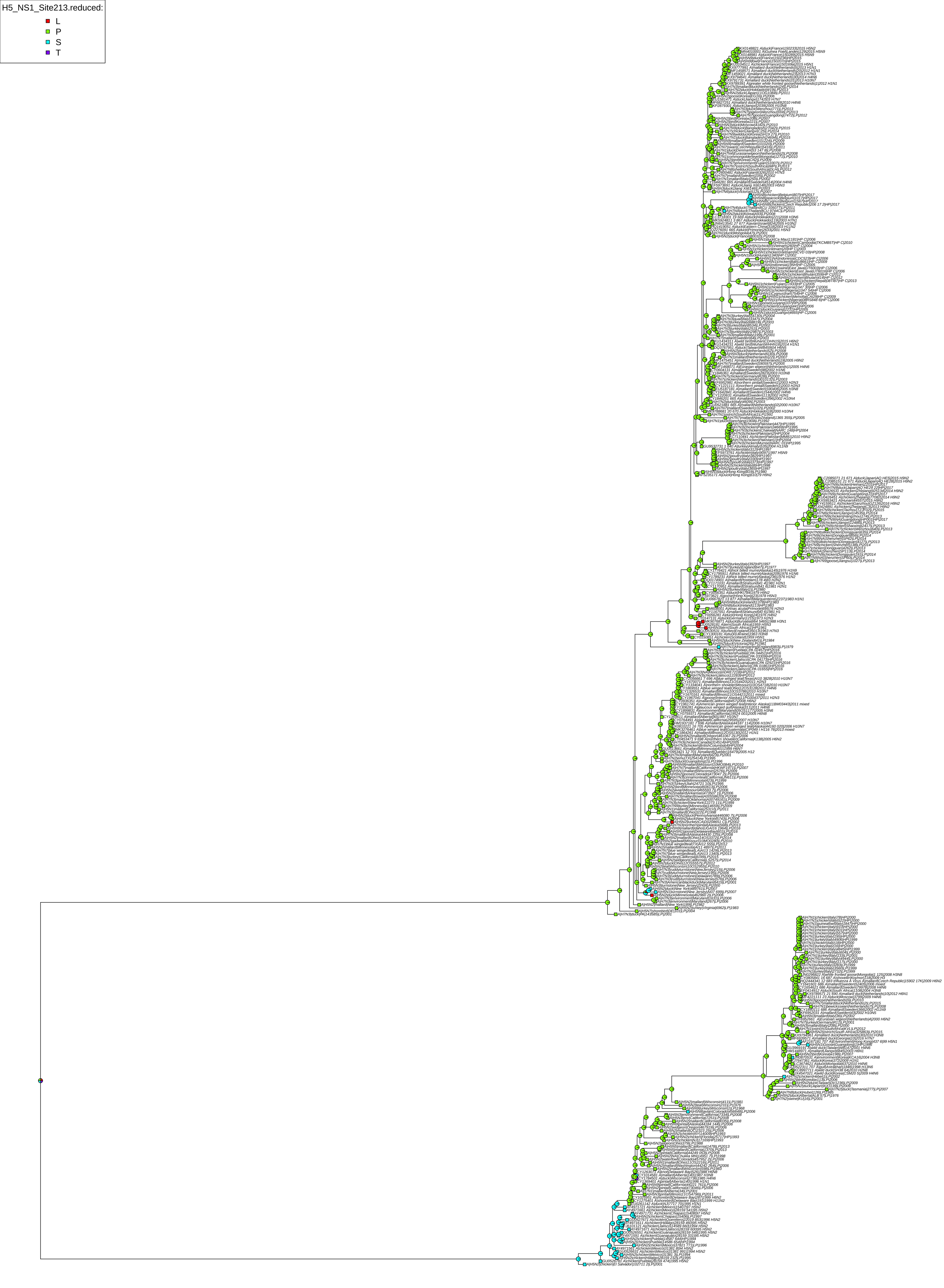
H5_NS1_Site213.reduced:
L
P
S
T

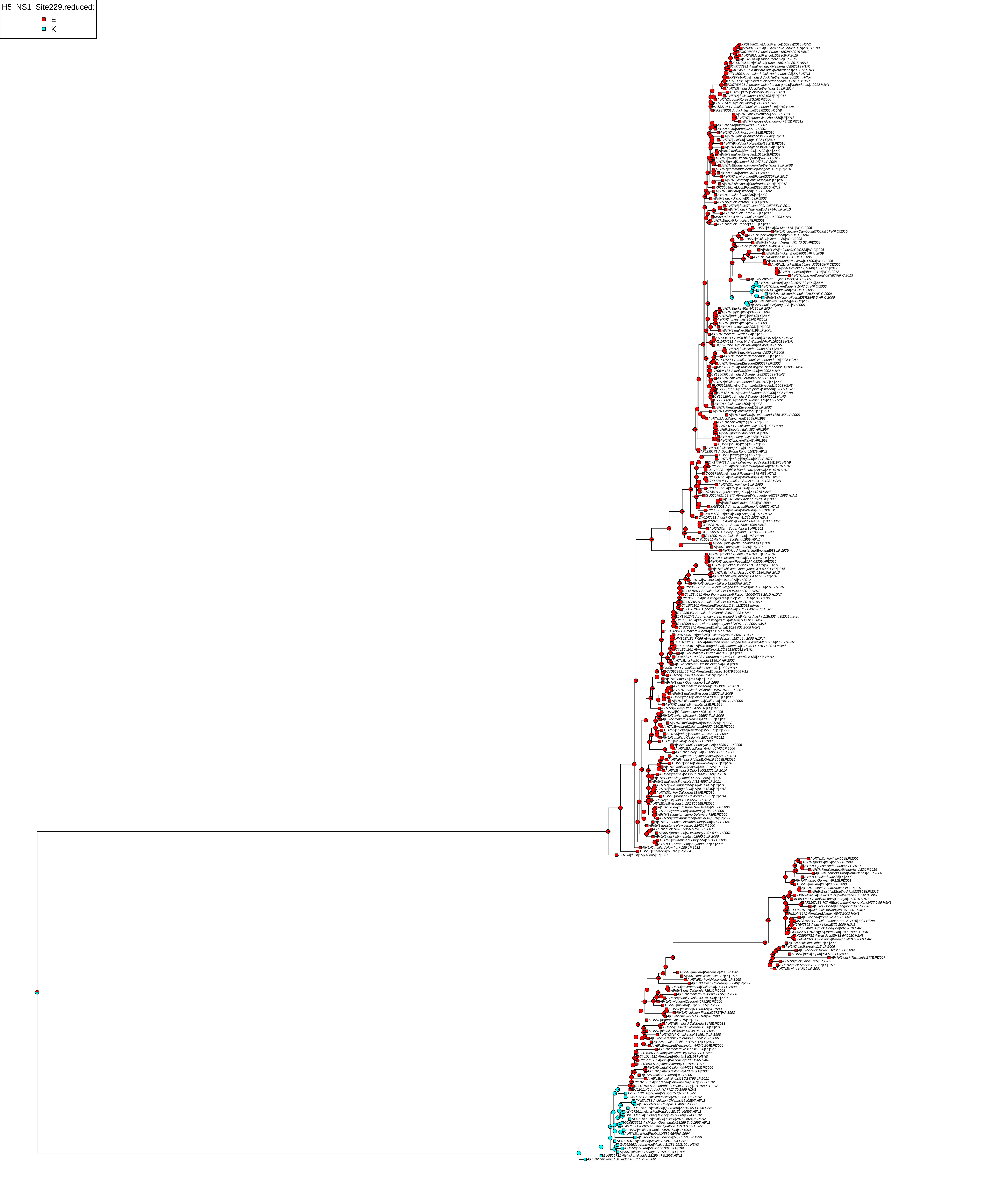

H5_NS1_Site229.reduced:
E
K

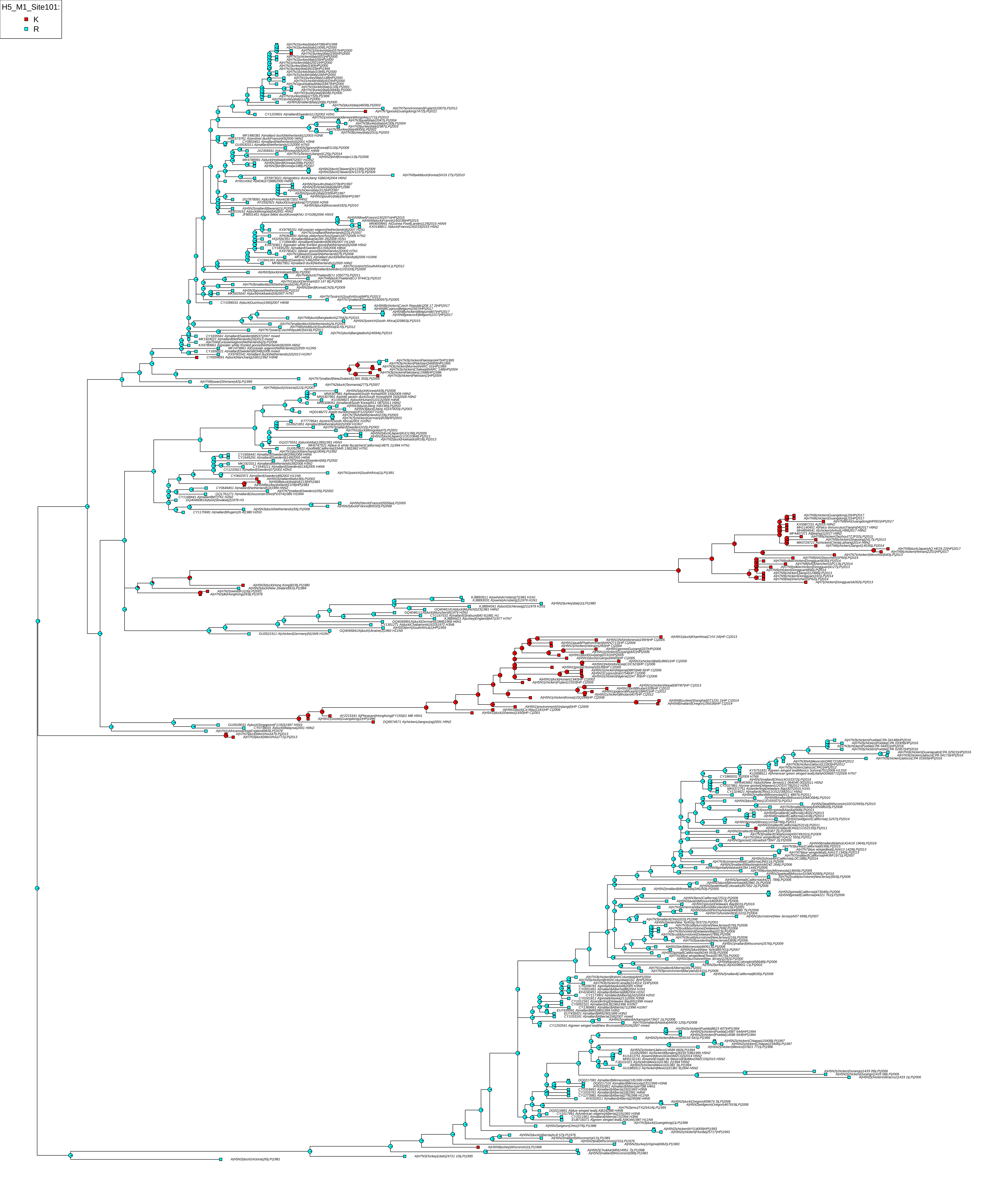
H5_M1_Site101:
K
R

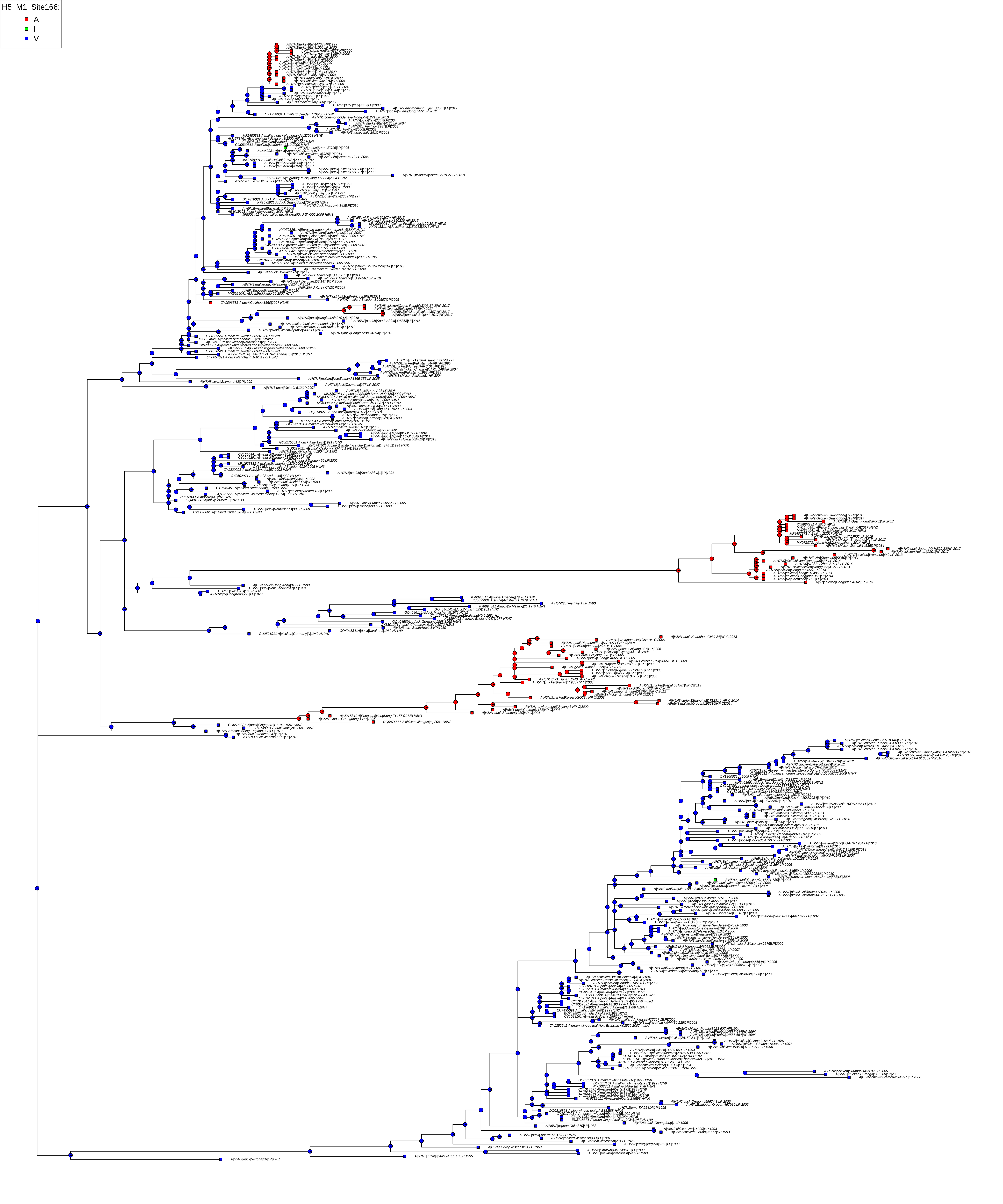
H5_M1_Site166:
A
I
V

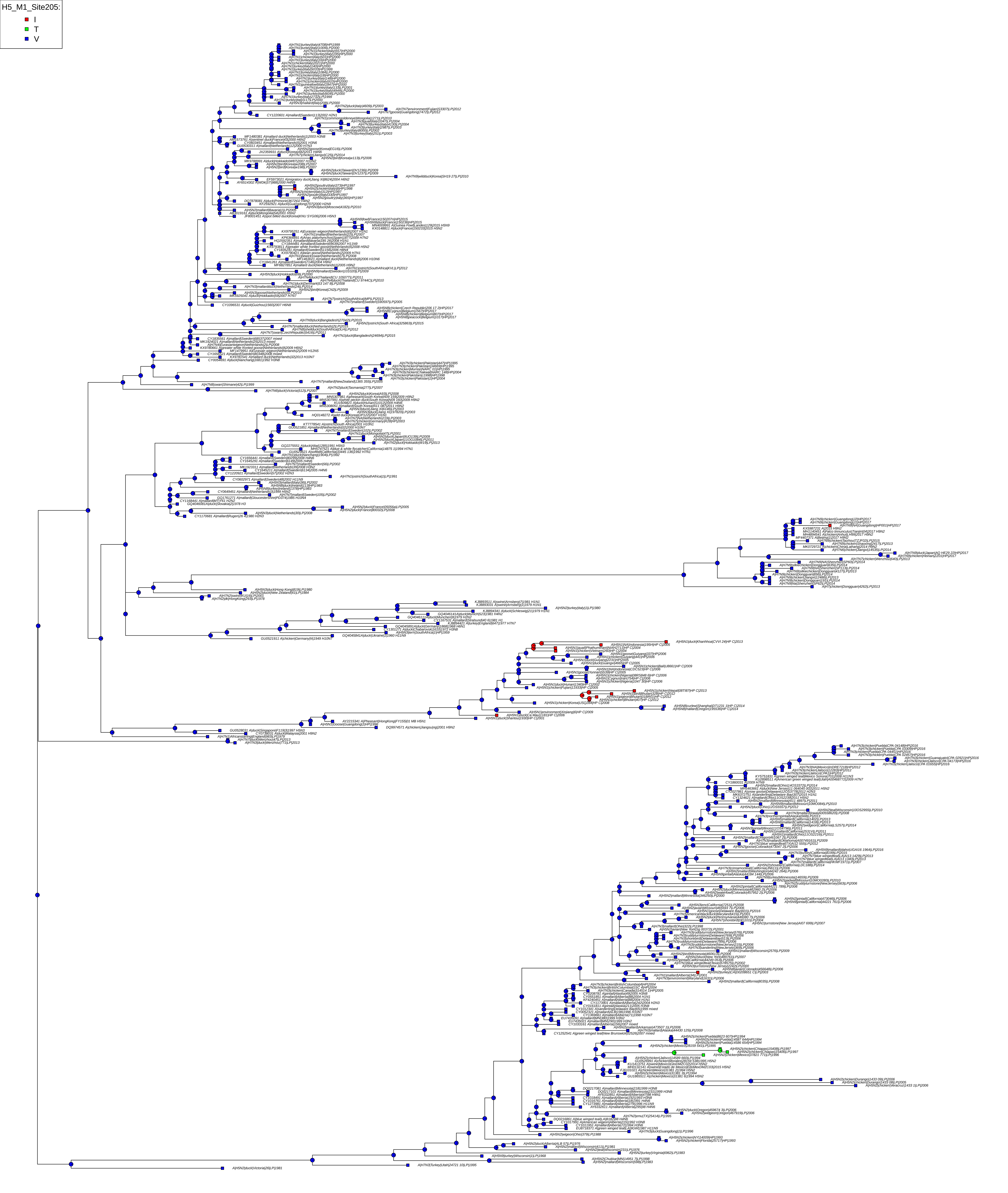
H5_M1_Site205:
I
T
V

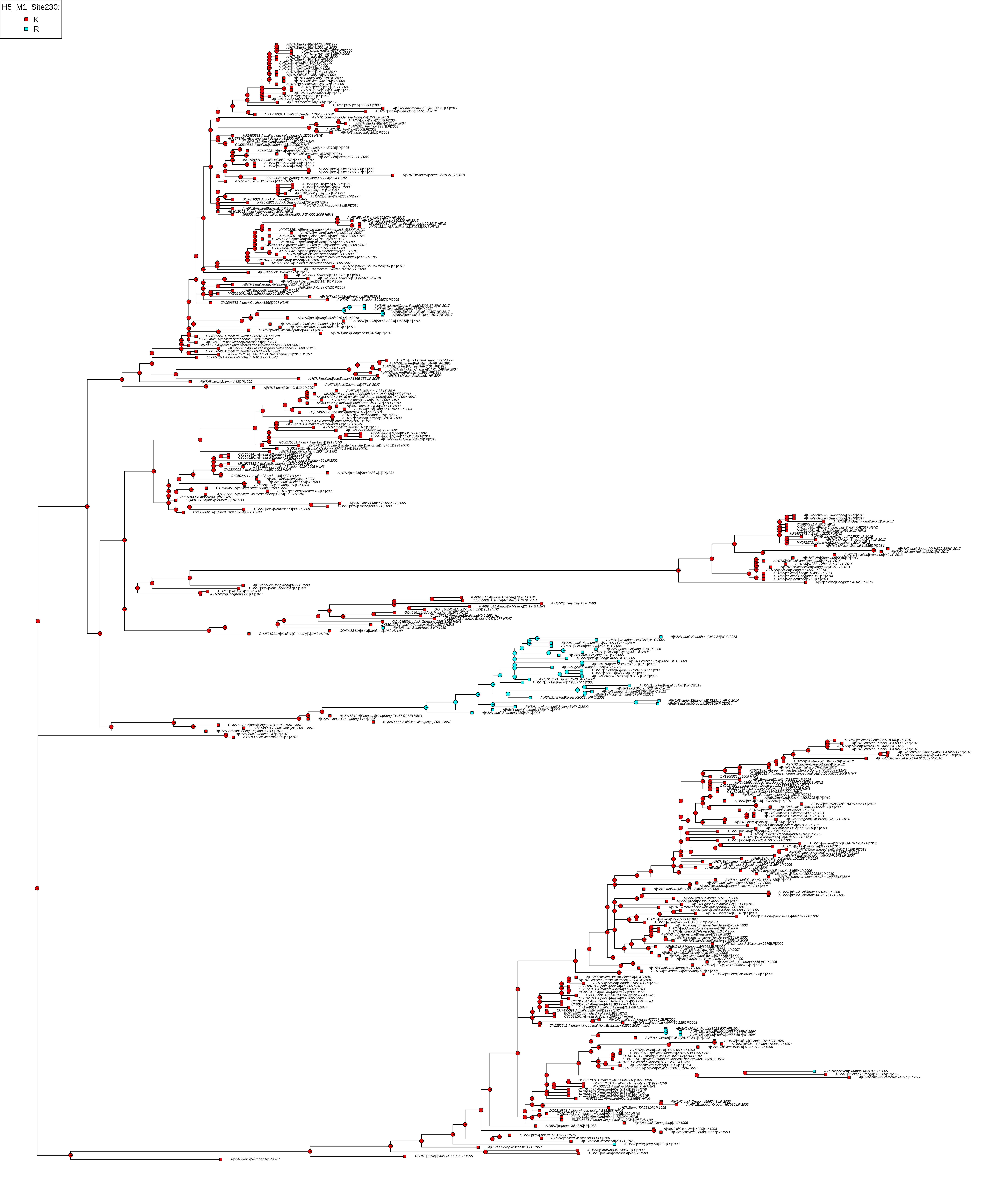

H5_M1_Site230:
K
R

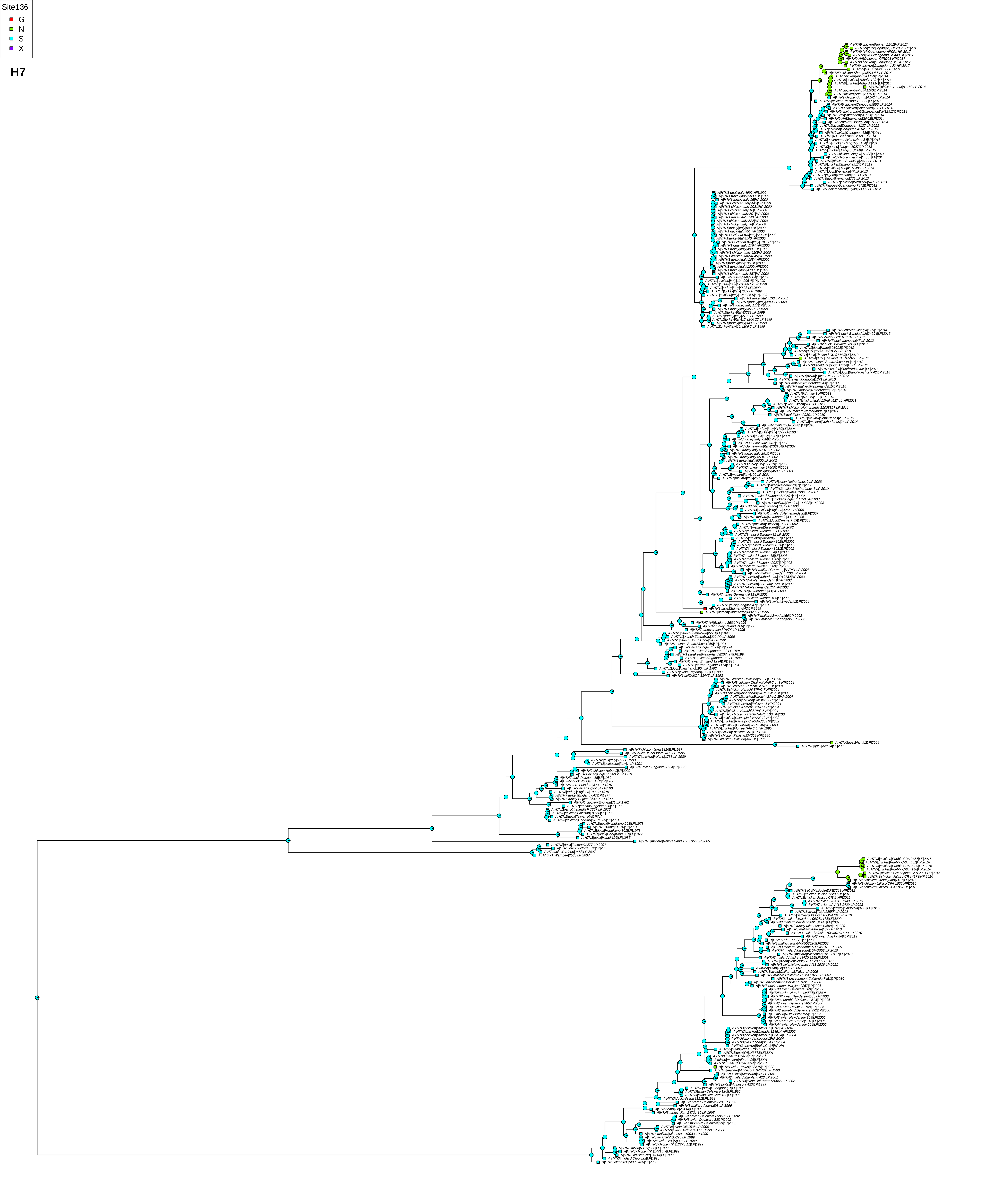
Site136
G
N
S
X
H7

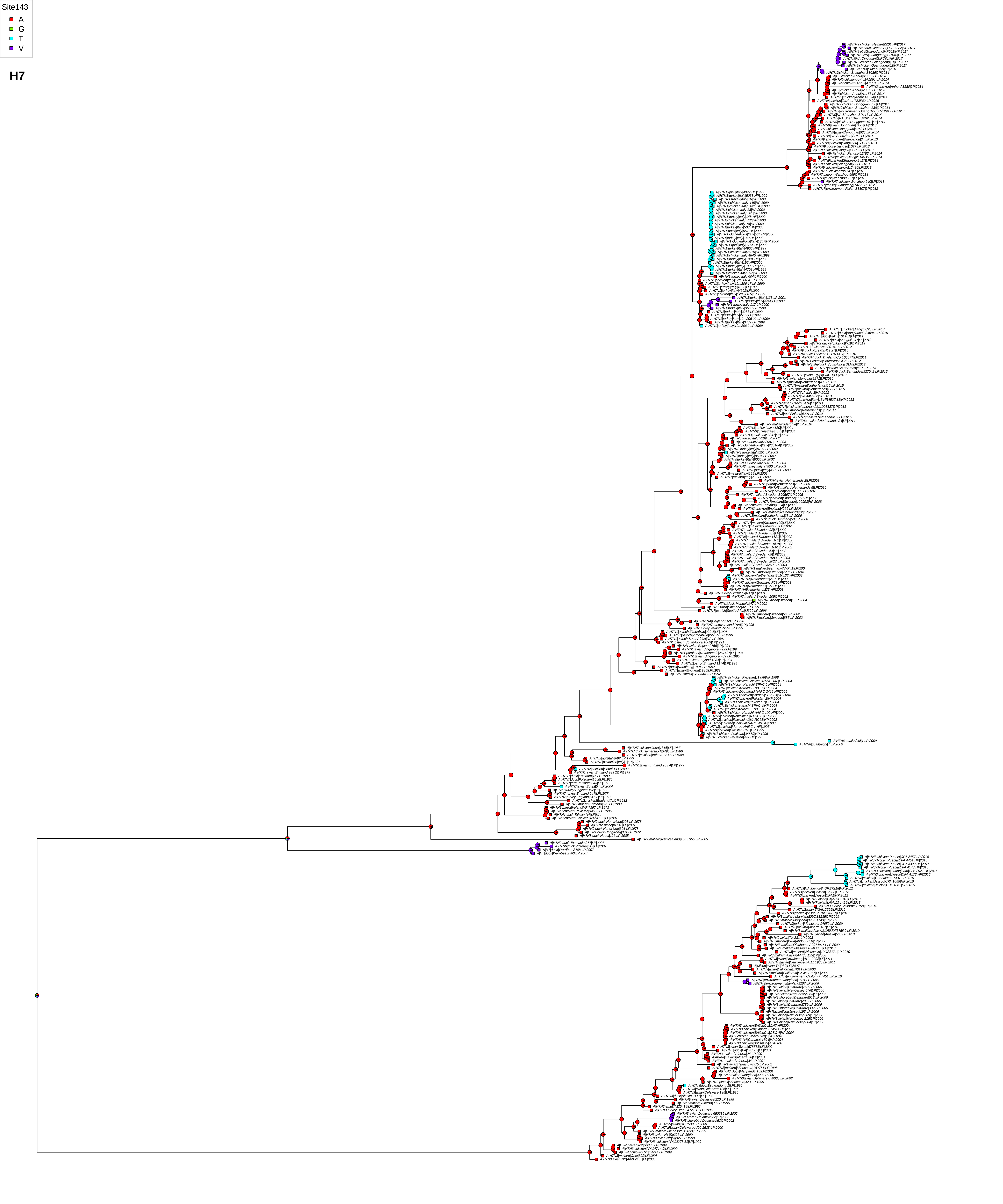
Site143
A
G
T
V
H7

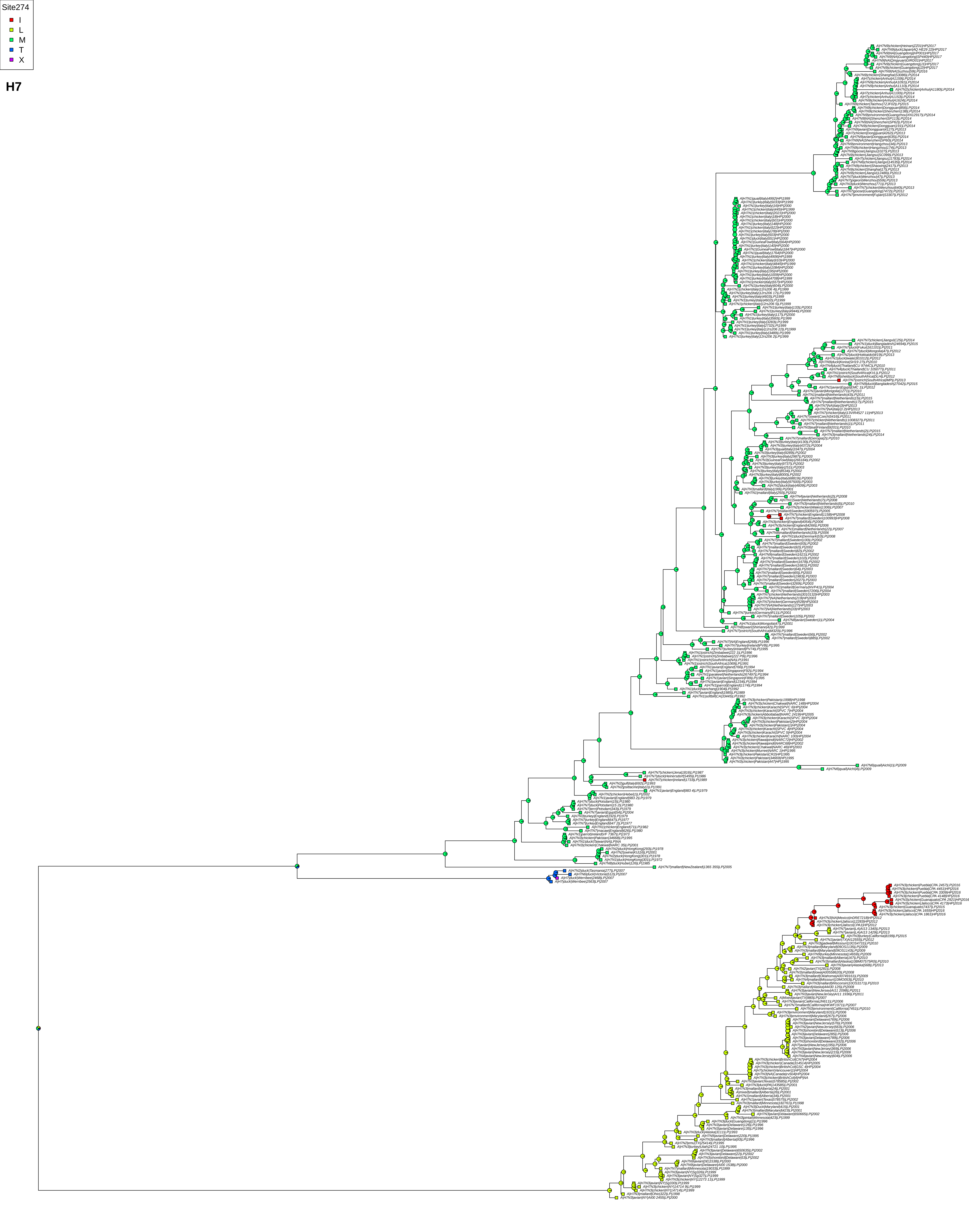

Site274
I
L
M
T
X
H7

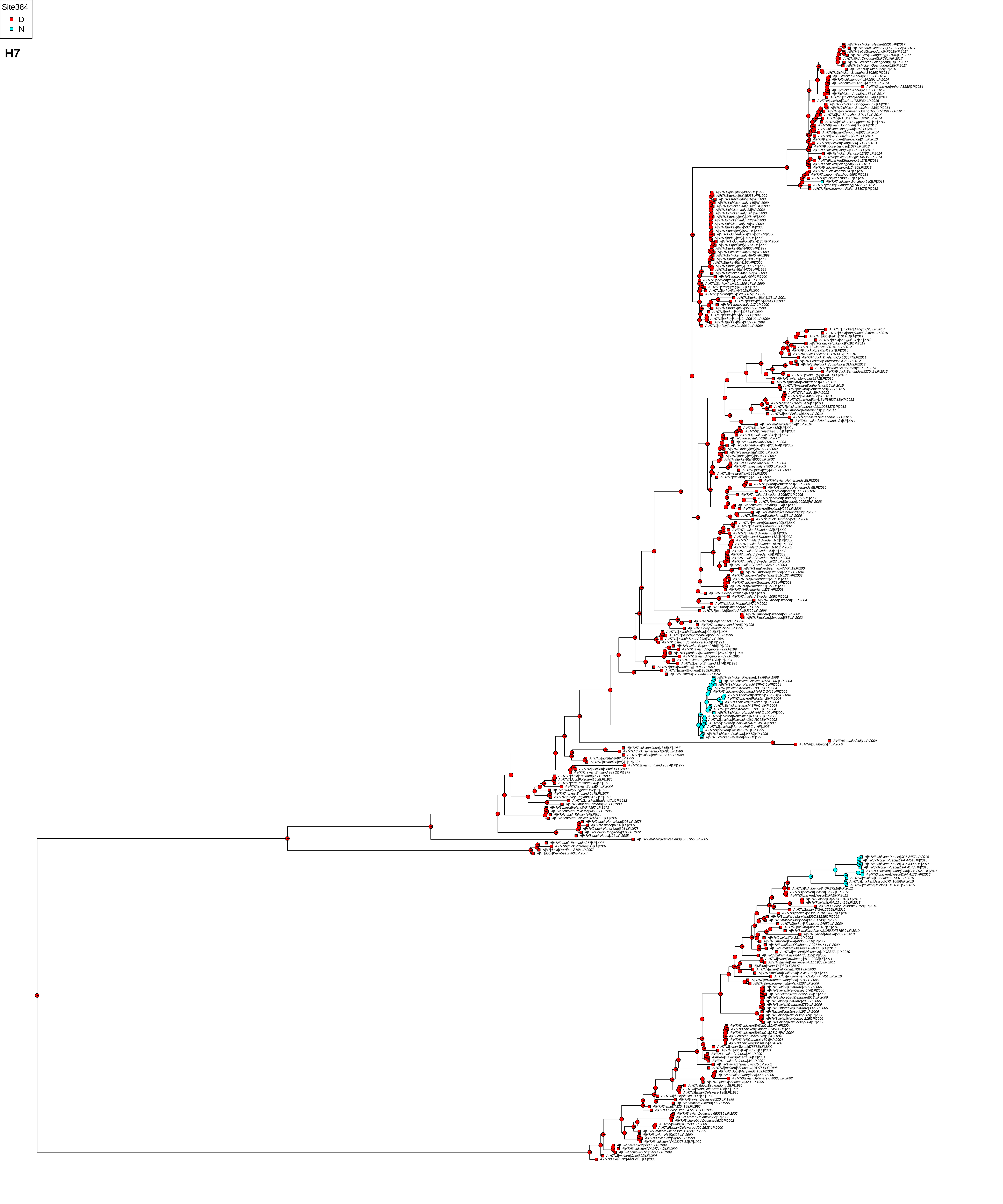
Site384
D
N
H7

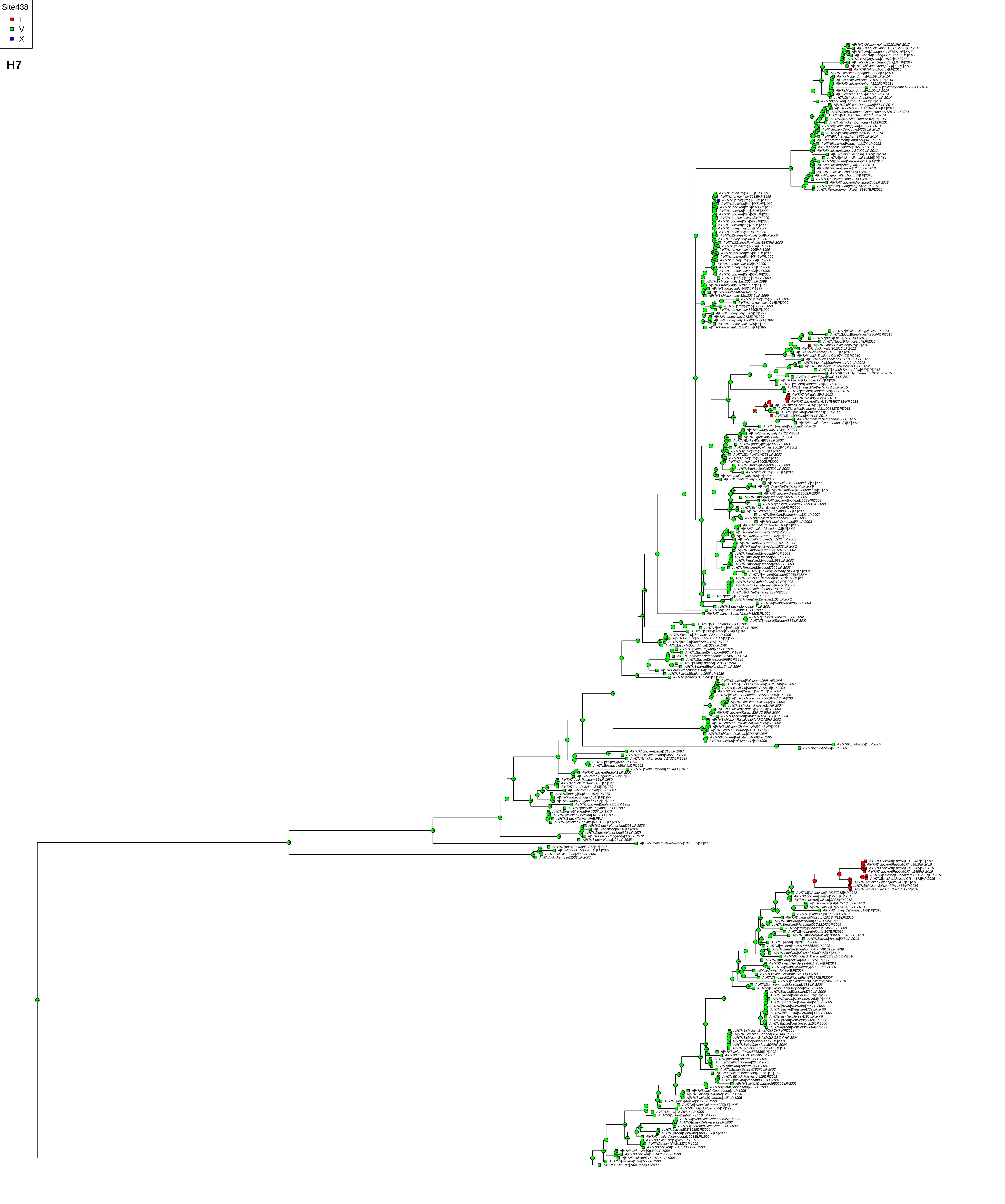
Site438
I
V
X
H7

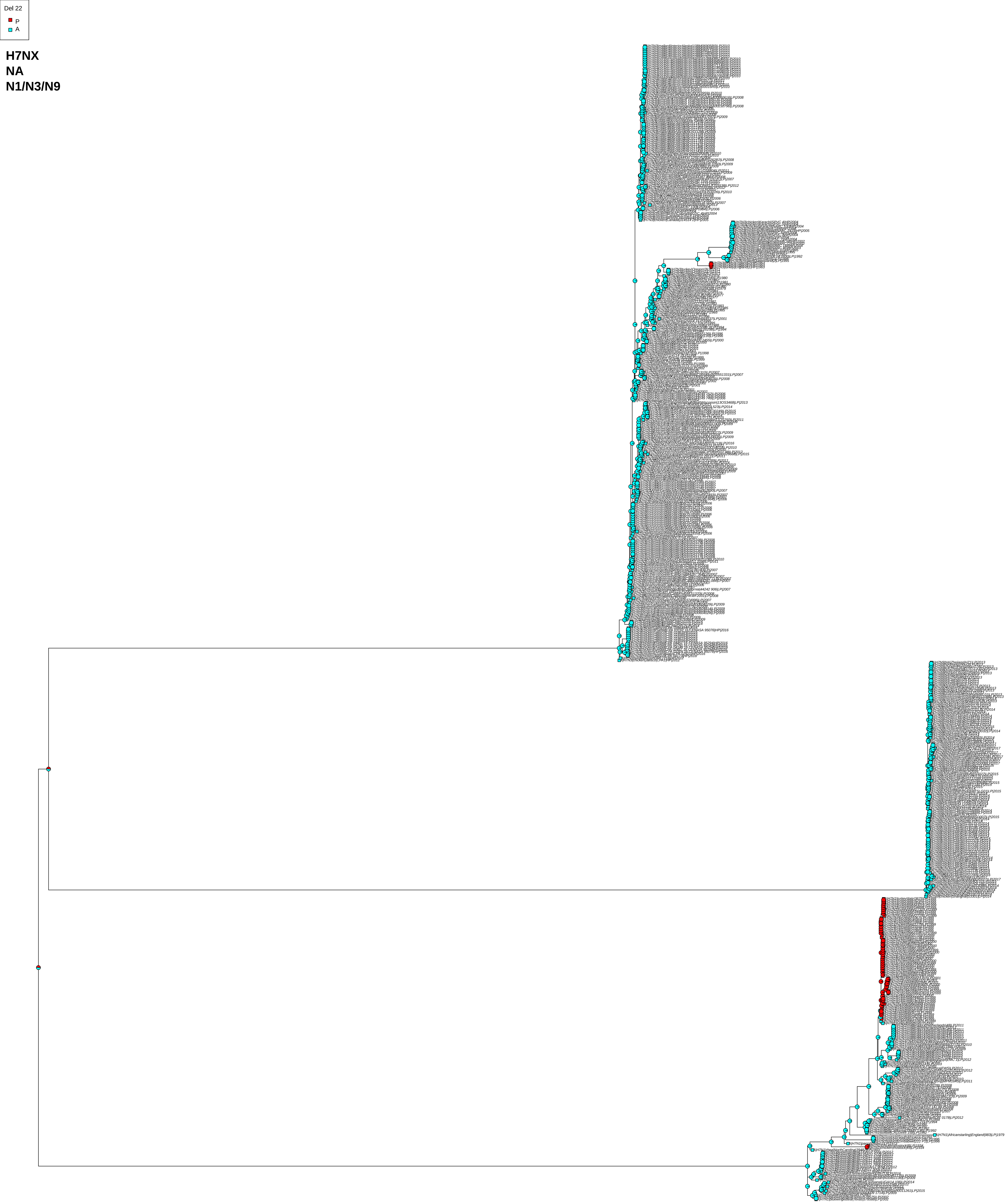
Del 22
P
A
H7NX
NA
N1/N3/N9

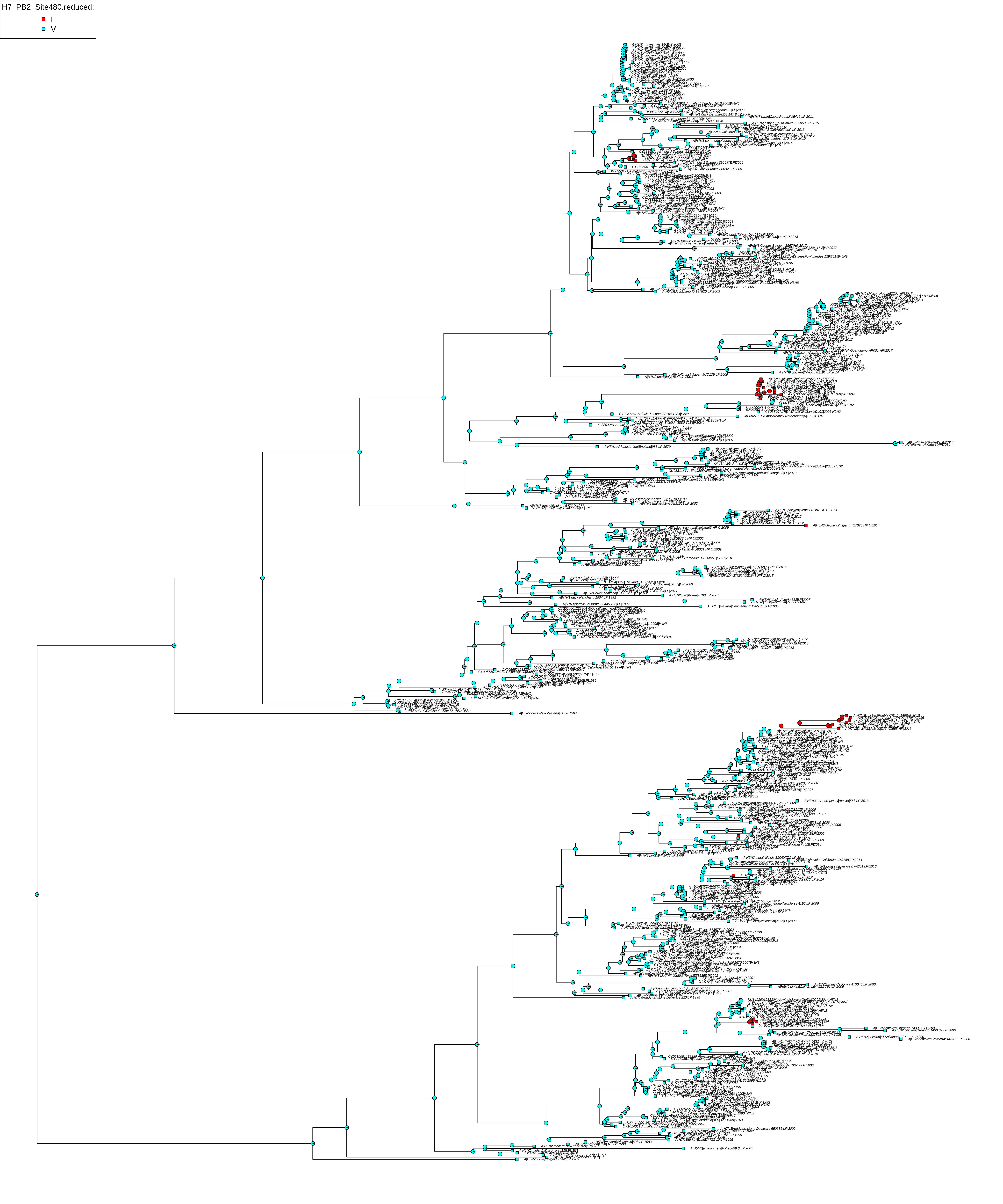
H7_PB2_Site480.reduced:
I
V

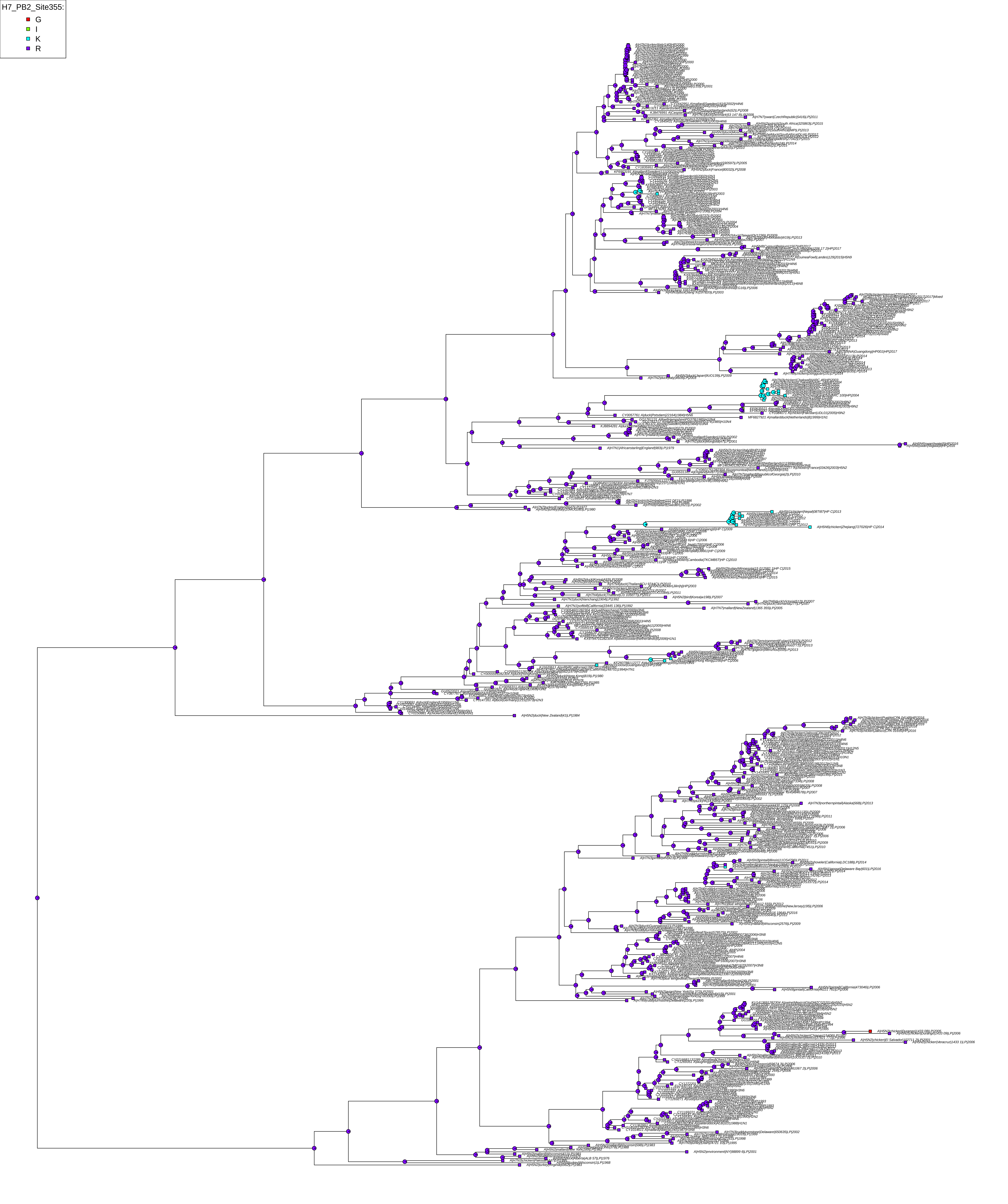

H7_PB2_Site355:
G
I
K
R

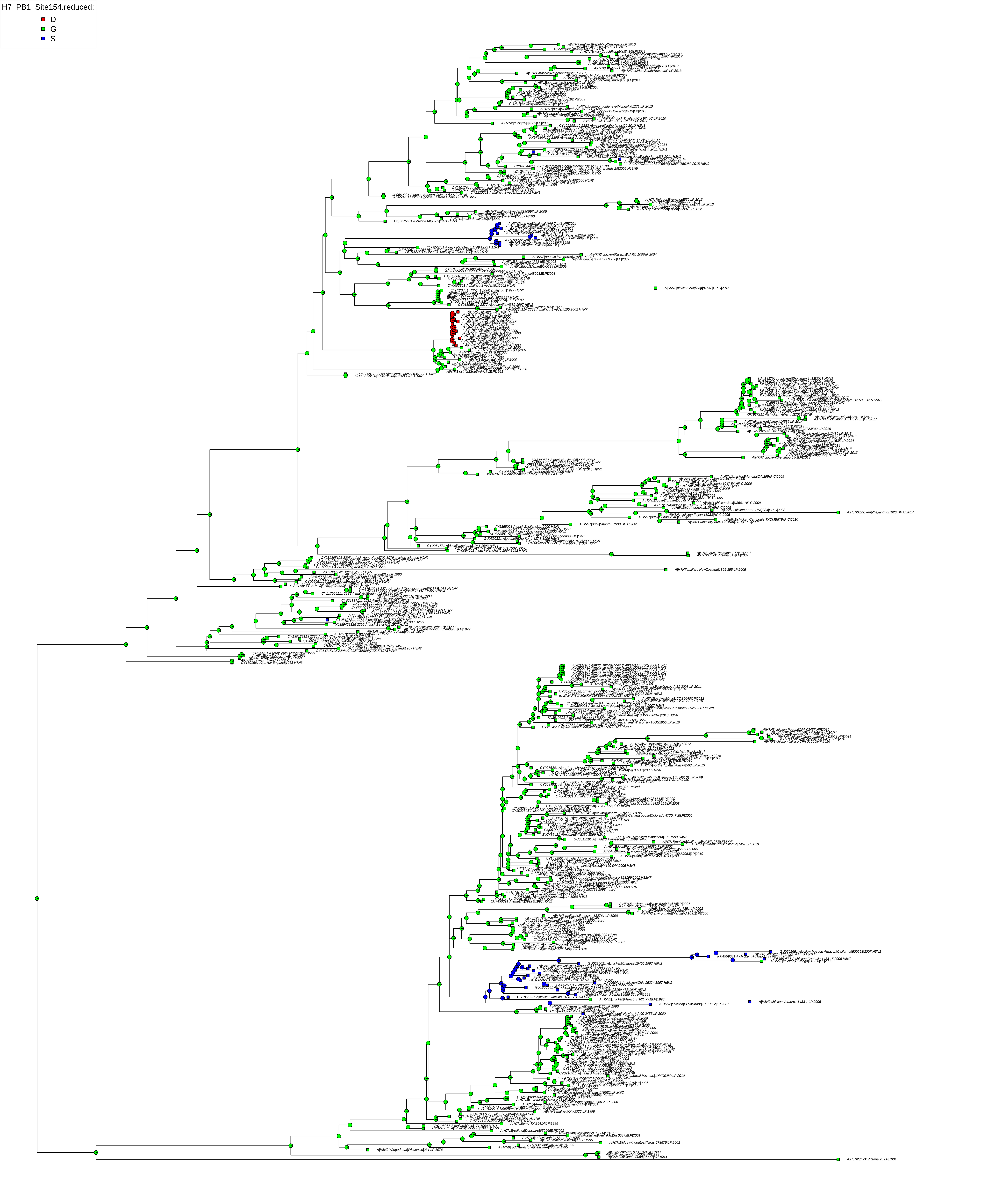
H7_PB1_Site154.reduced:
D
G
S

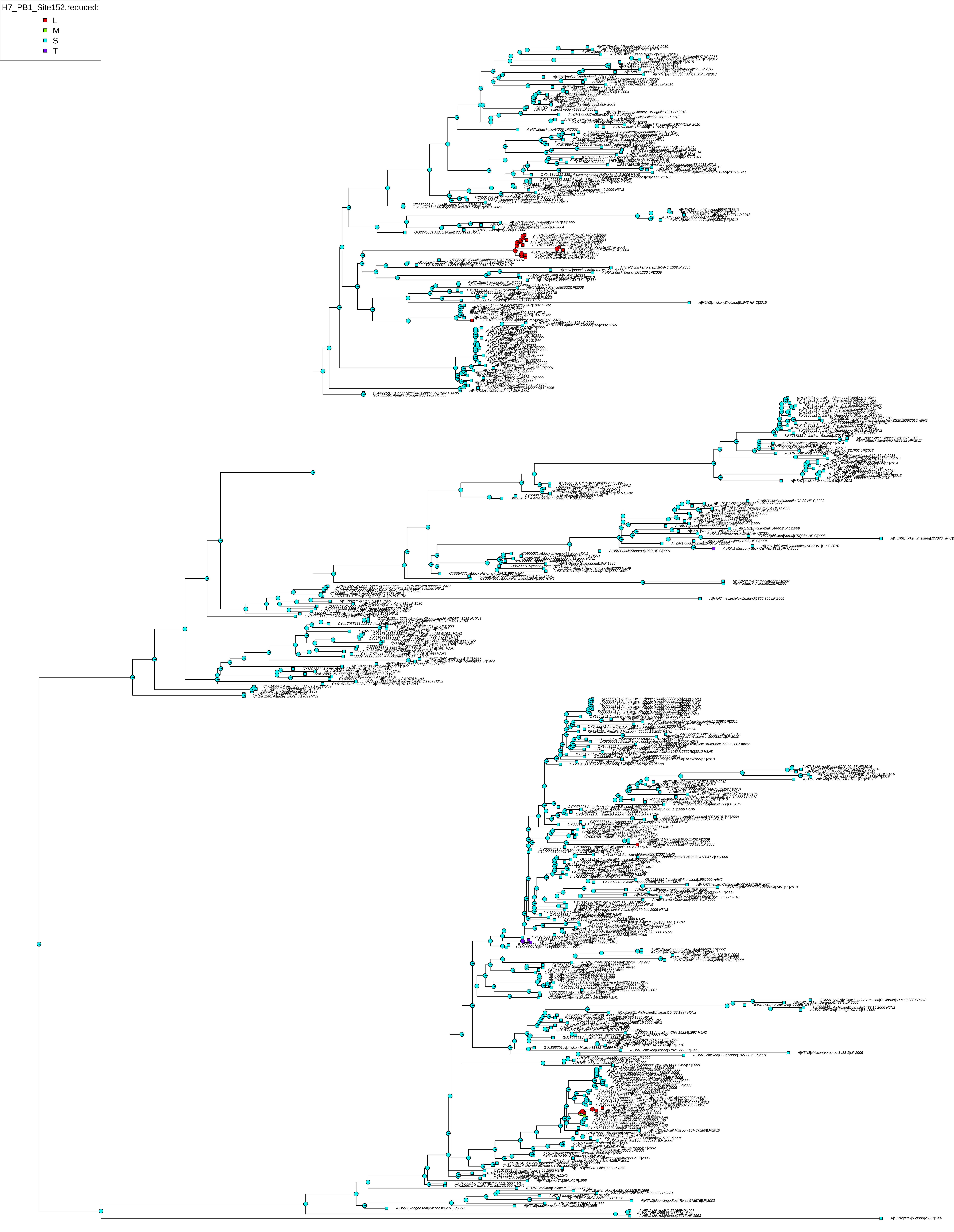

H7_PB1_Site152.reduced:
L
M
S
T

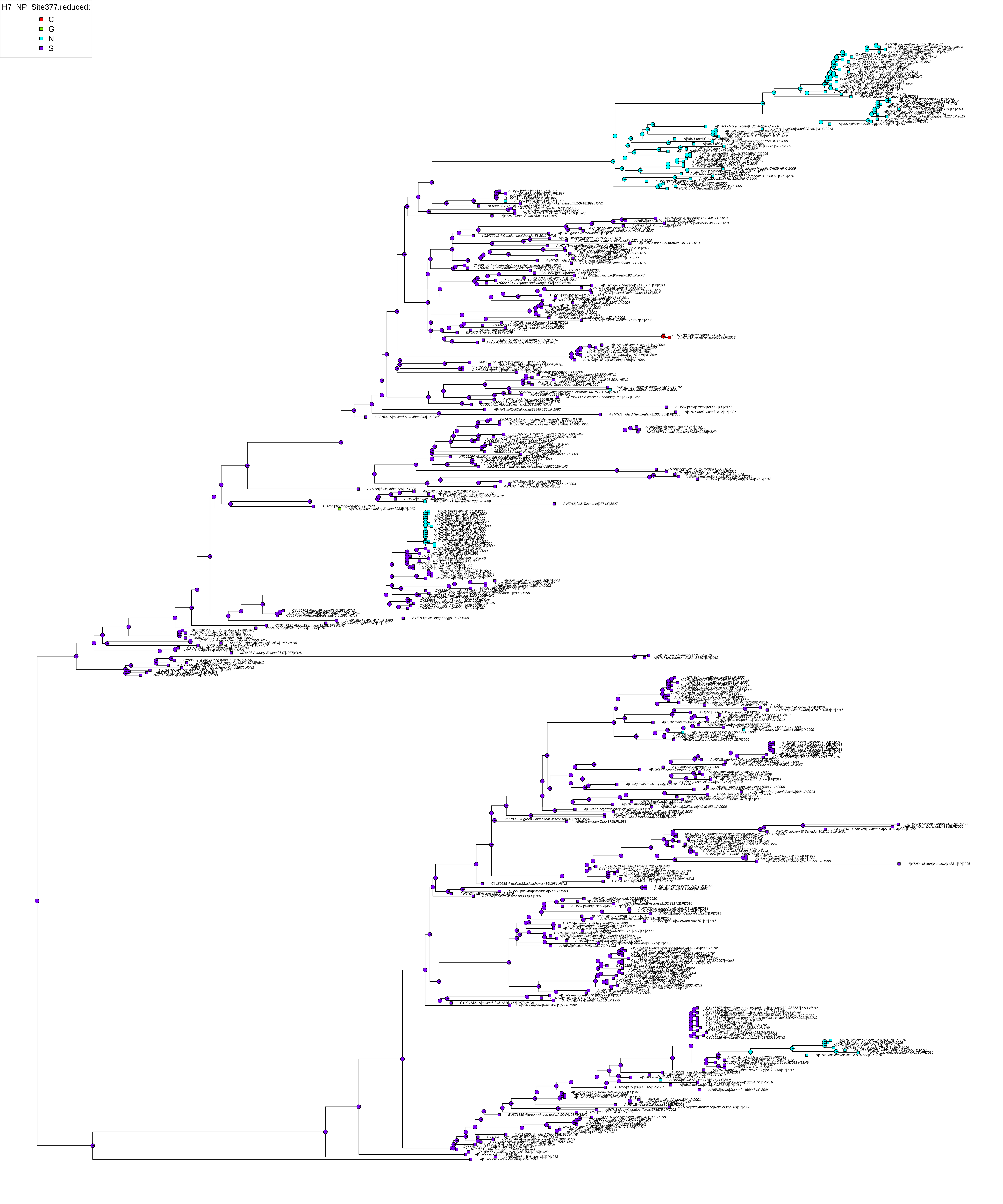

H7_NP_Site377.reduced:
C
G
N
S

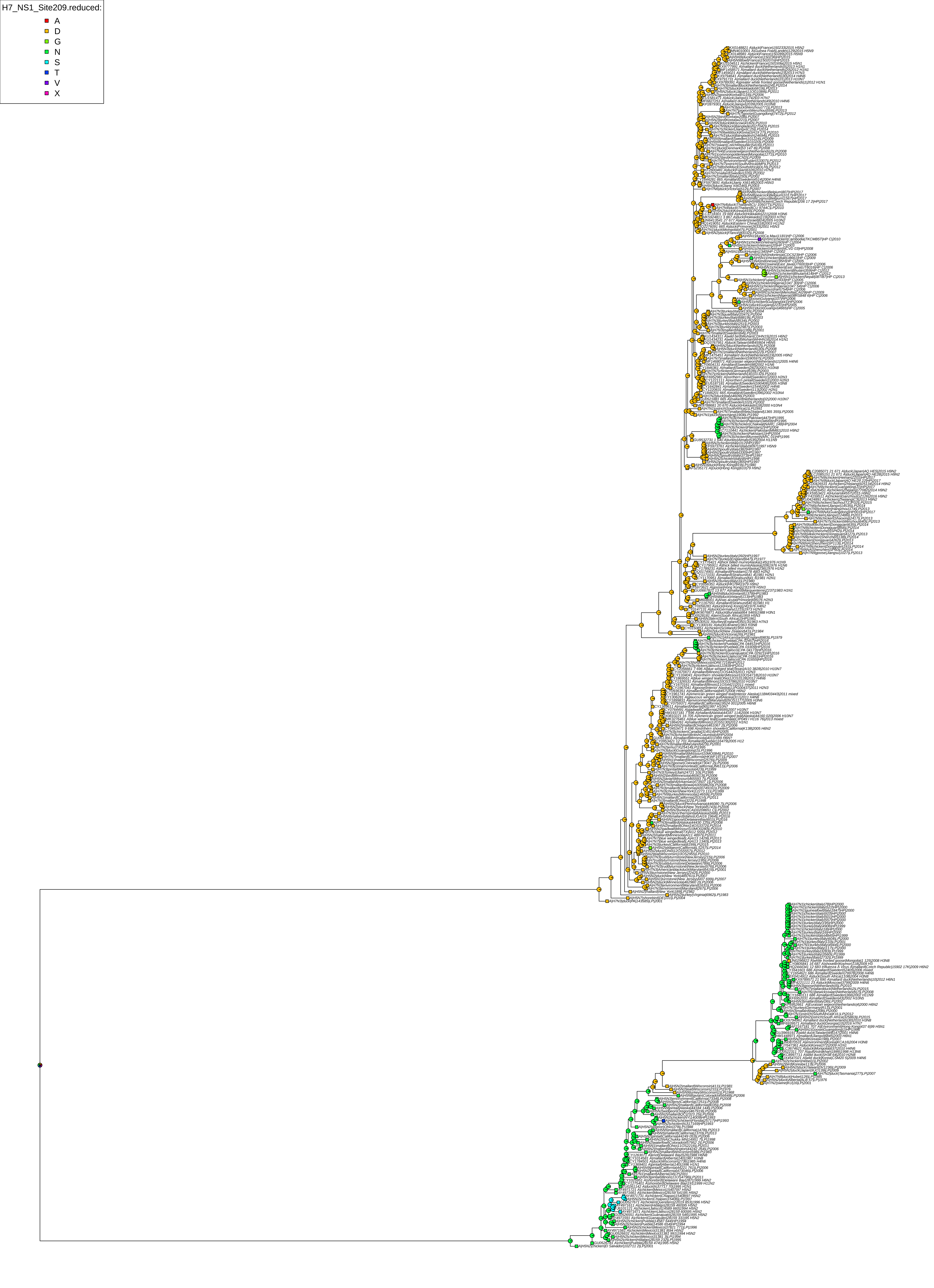
H7_NS1_Site209.reduced:
A
D
G
N
S
T
V
X

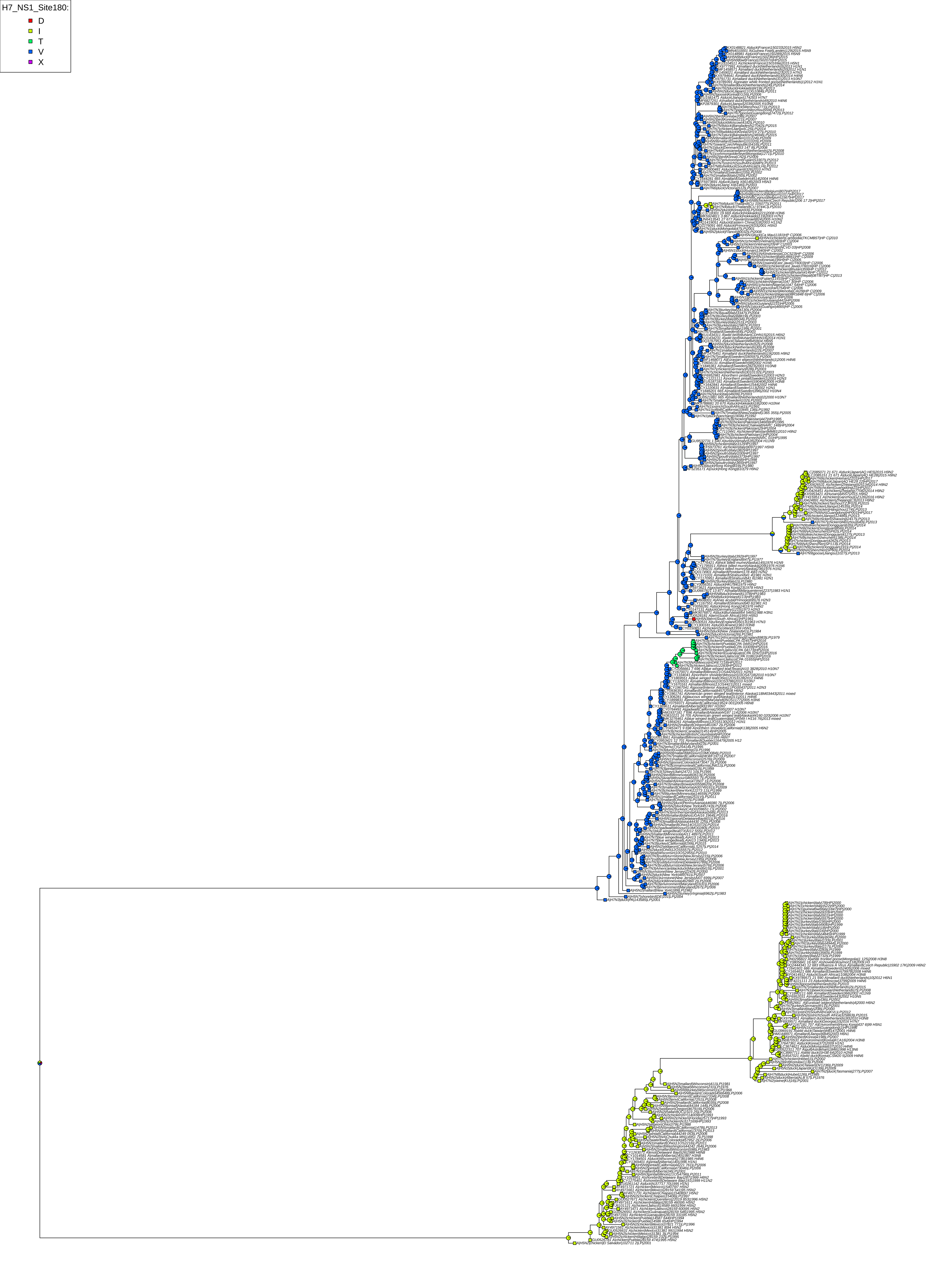
H7_NS1_Site180:
D
I
T
V
X

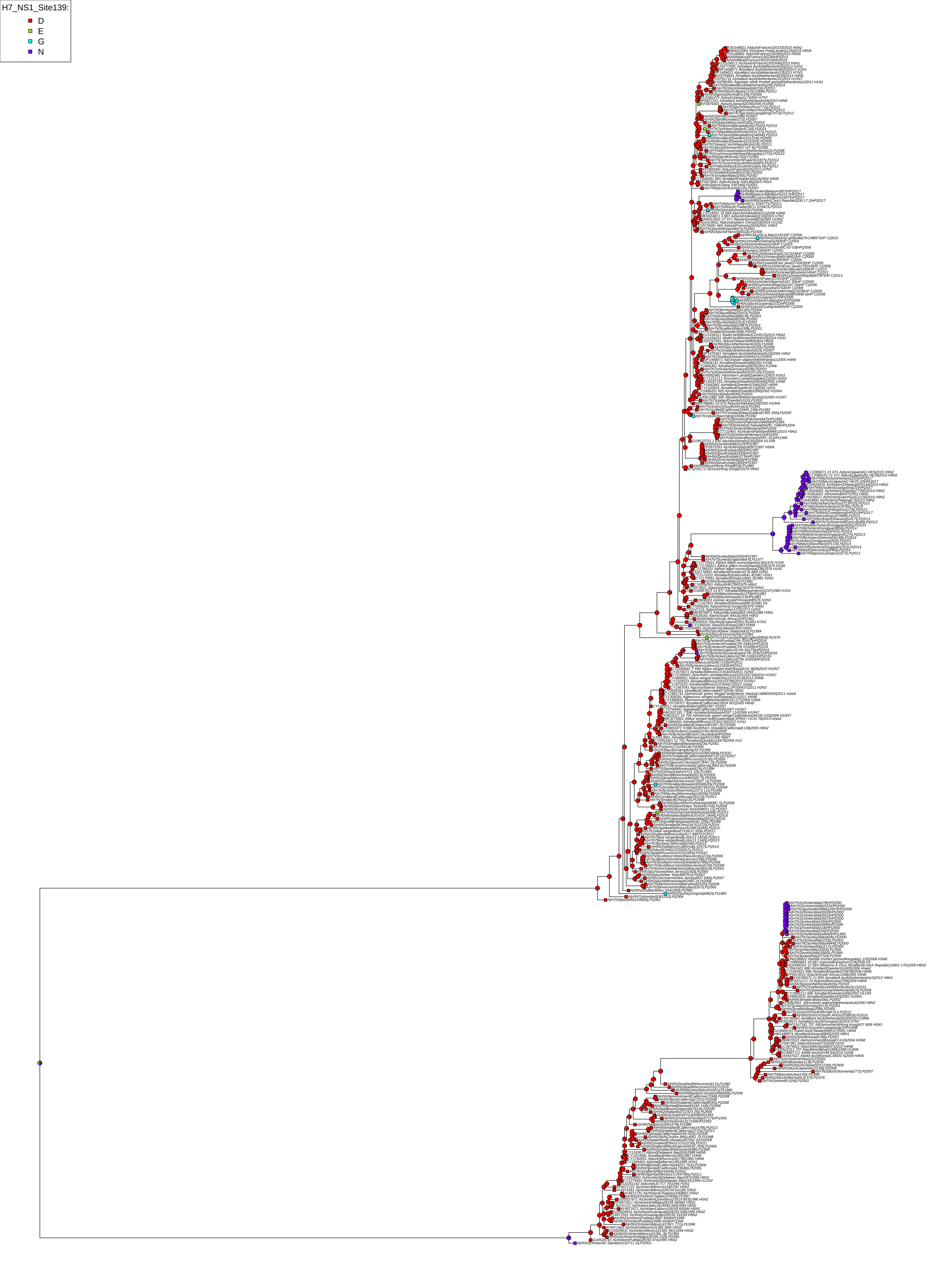
H7_NS1_Site139:
D
E
G
N

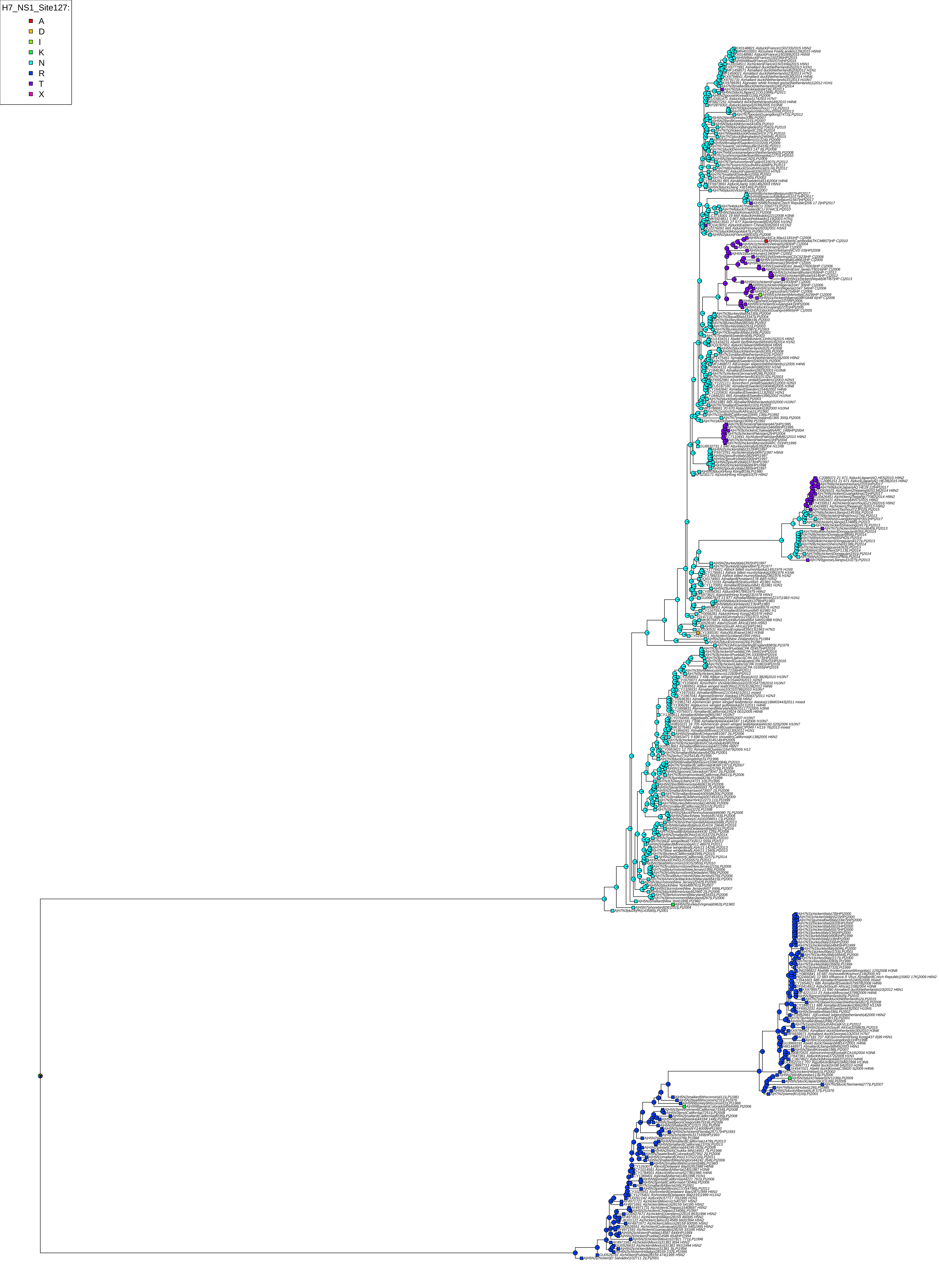
H7_NS1_Site127:
A
D
I
K
N
R
T
X

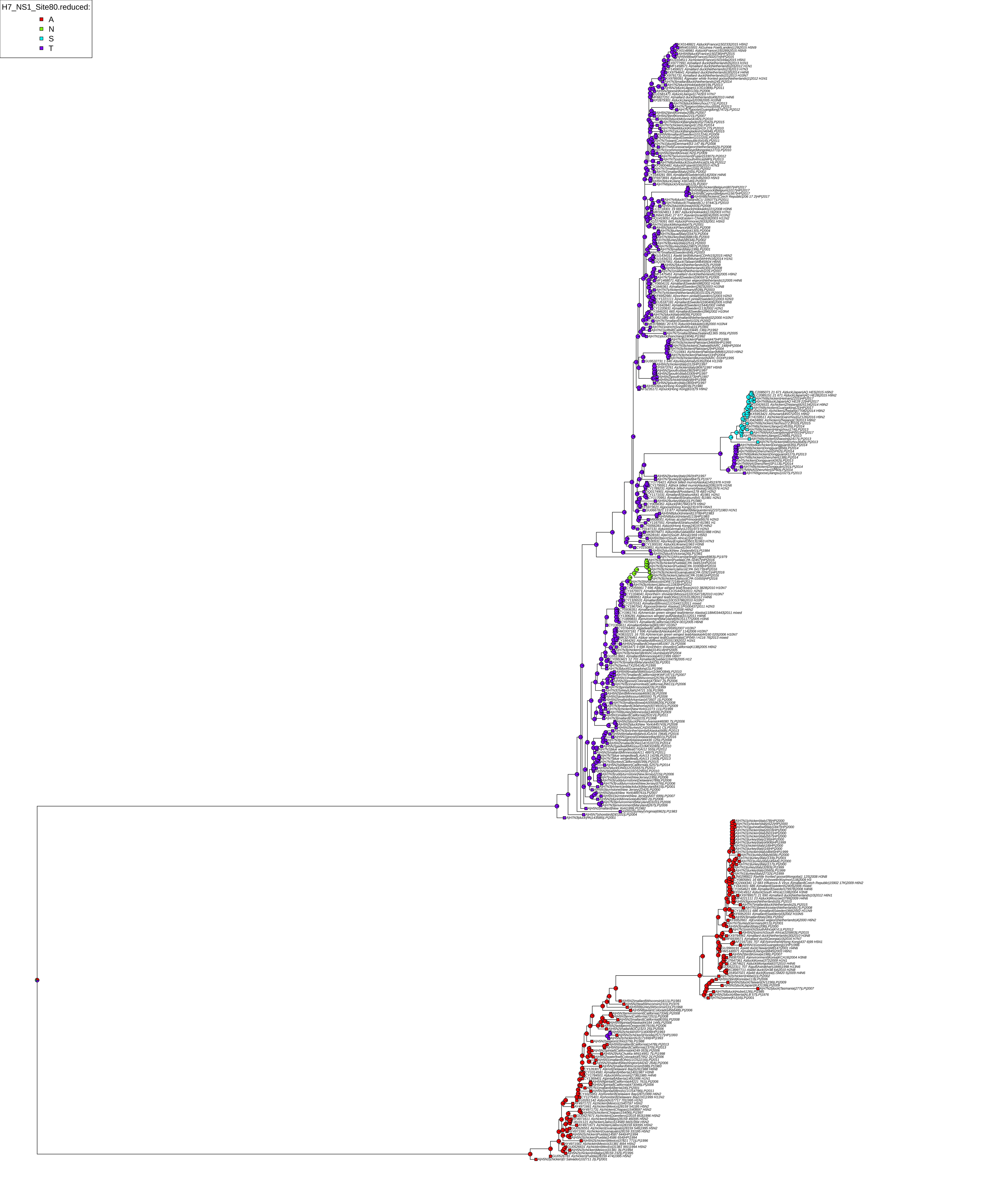
H7_NS1_Site80.reduced:
A
N
S
T
