# Supplementary material for: Parallel evolution in the emergence of highly pathogenic avian influenza A viruses
Source: Nat Commun. 2020 Nov 2;11:5511. doi: 10.1038/s41467-020-19364-x (PMC7608645; doi:10.1038/s41467-020-19364-x)
Supplement: Supplementary file 6 — Supplementary Data 3 [file 41467_2020_19364_MOESM6_ESM.pdf]

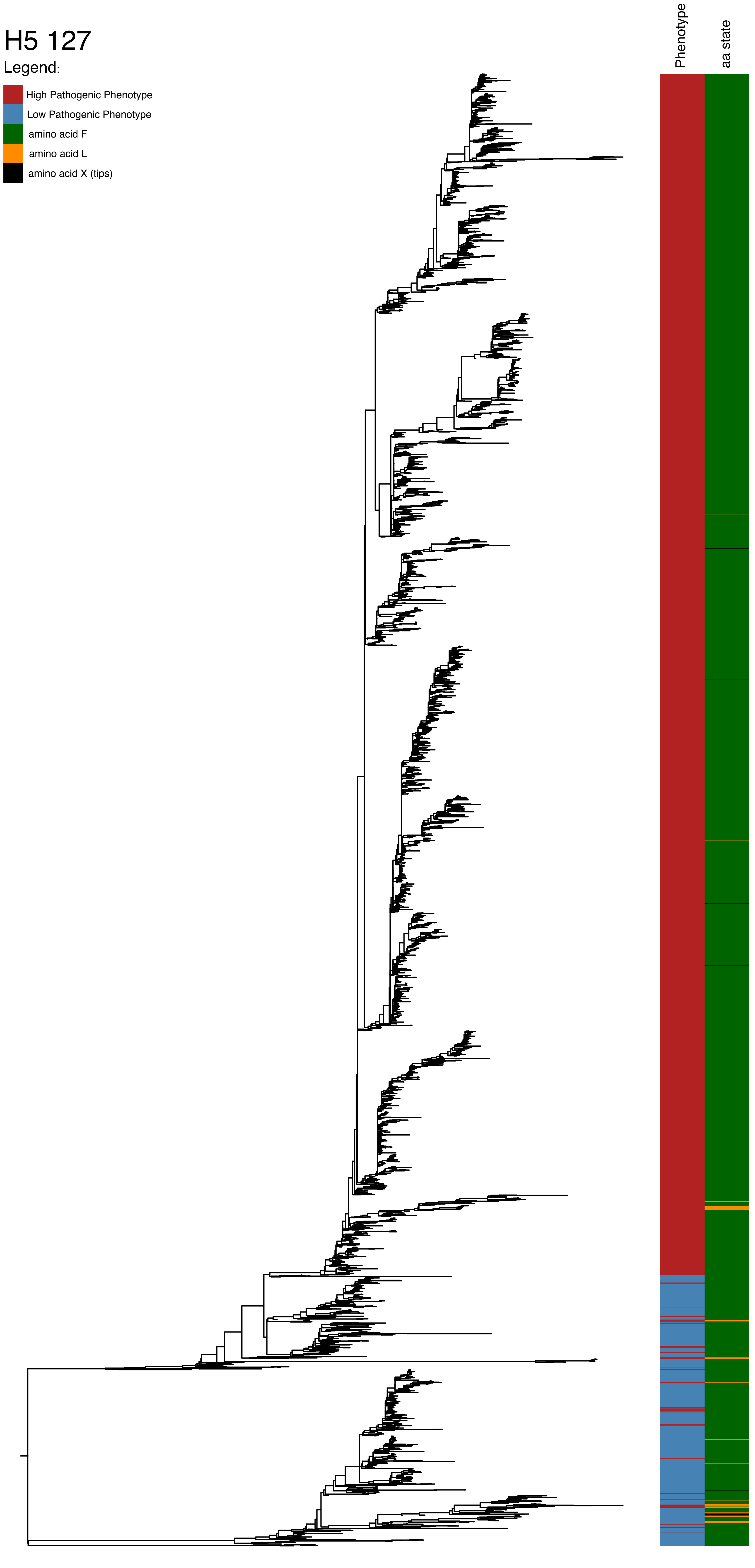
H5 127
Legend:
High Pathogenic Phenotype
Low Pathogenic Phenotype
amino acid F
amino acid L
amino acid X (tips)
Phenotype
aa state

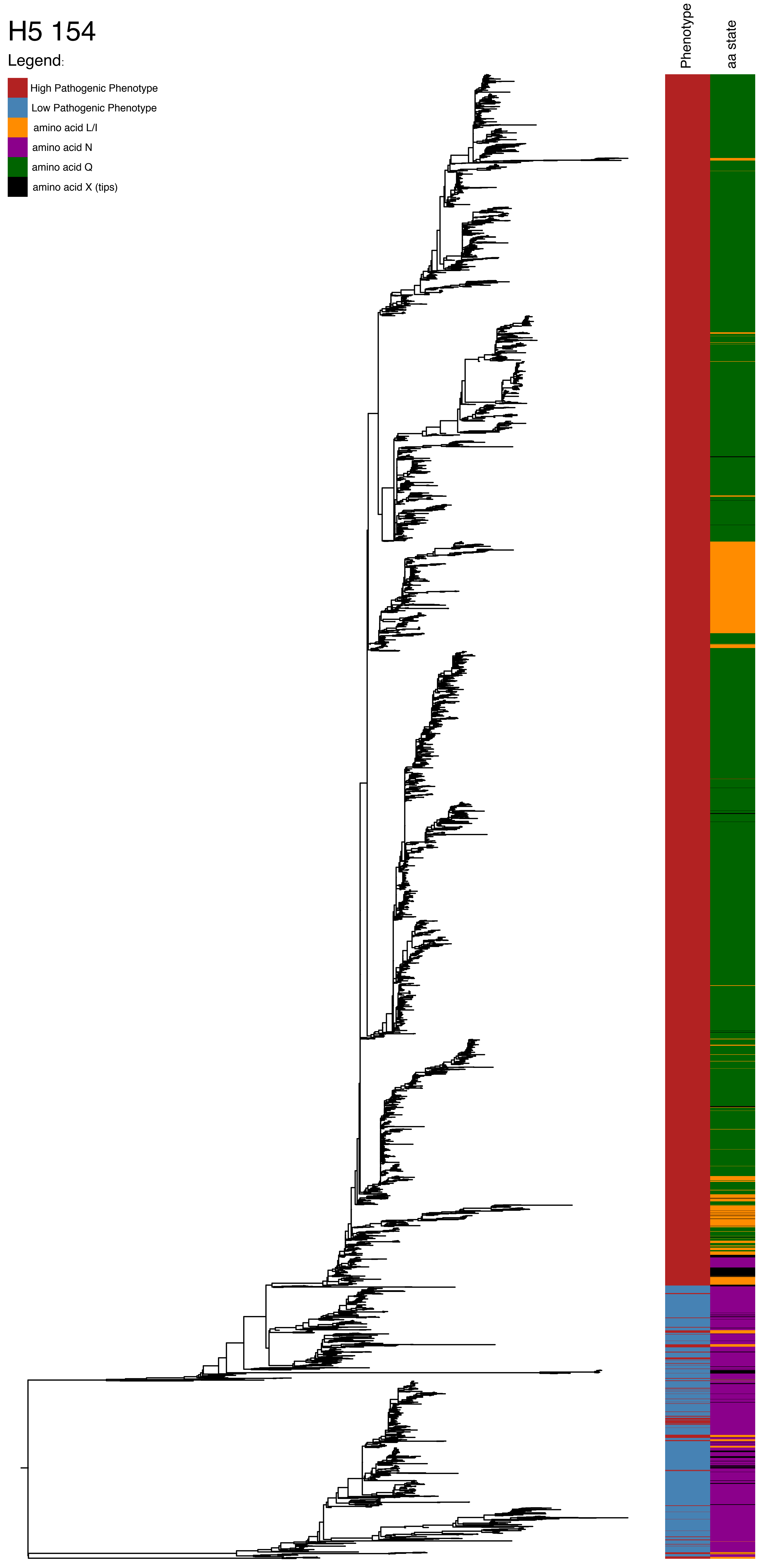
H5 154
Legend:
High Pathogenic Phenotype
Low Pathogenic Phenotype
amino acid L/I
amino acid N
amino acid Q
amino acid X (tips)
Phenotype
aa state

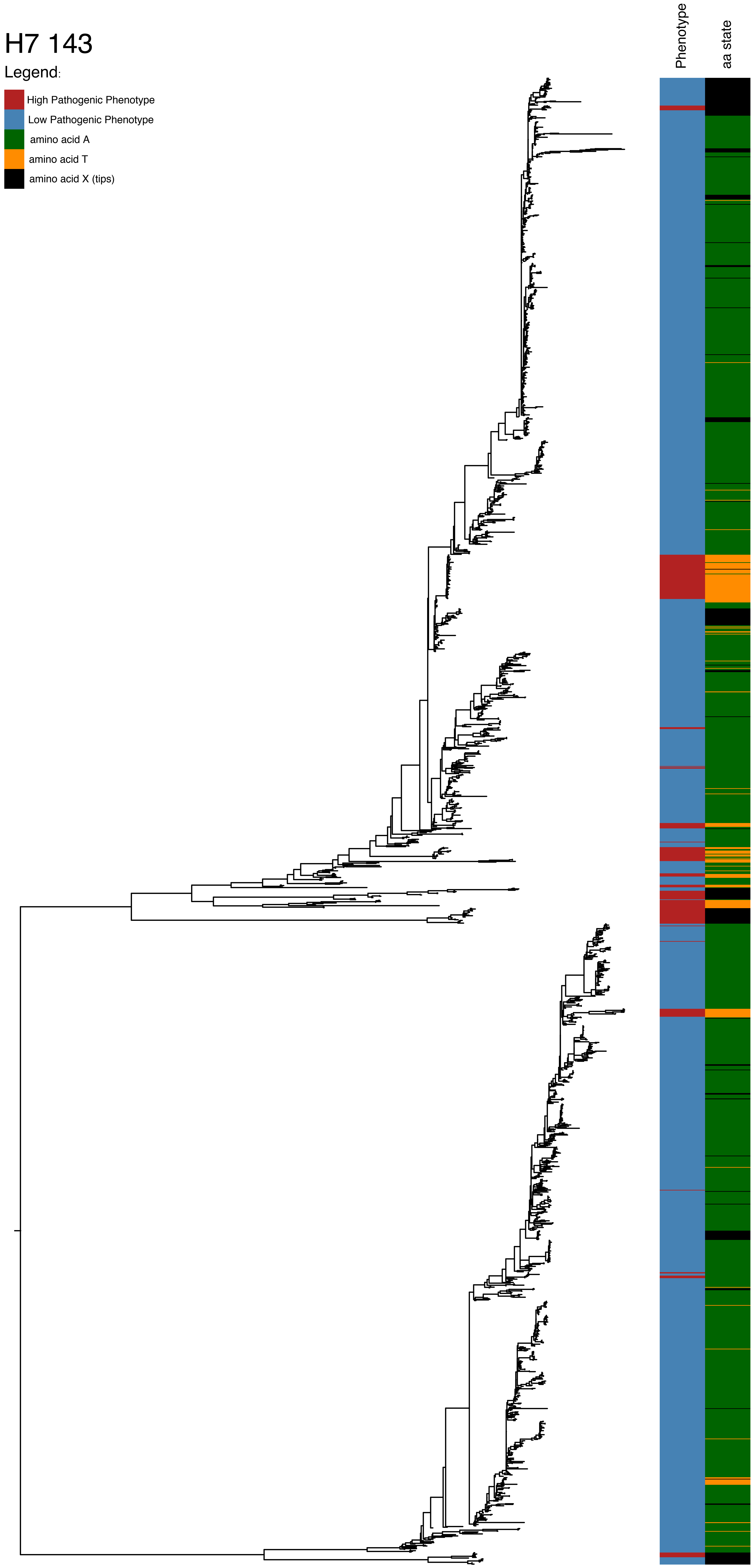
H7 143
Legend:
High Pathogenic Phenotype
Low Pathogenic Phenotype
amino acid A
amino acid T
amino acid X (tips)
Phenotype
aa state
